# Genomic Variation Underpins Genetic Divergence and Differing Salt Resilience in *Sesbania bispinosa*

**DOI:** 10.1002/advs.202502600

**Published:** 2025-05-29

**Authors:** Gai Huang, Xiaofei Wang, Chengli Liu, Kaixuan He, Xiu‐Li Hou, Haofei Luo, Shuaibin Zhang, Changqing You, Yajun Jia, Fuqiang Wang, Xianwei Song, Guodao Liu, Xian Deng, Xiaofeng Cao

**Affiliations:** ^1^ State Key Laboratory of Seed Innovation Institute of Genetics and Developmental Biology Chinese Academy of Sciences Beijing 100101 China; ^2^ Laboratory of Advanced Breeding Technologies Institute of Genetics and Developmental Biology Chinese Academy of Sciences Beijing 100101 China; ^3^ School of Tropical Agriculture and Forestry Hainan University Haikou Hainan 570288 China; ^4^ Hainan Seed Industry Laboratory Sanya 572024 China; ^5^ Tropical Crops Genetic Resources Institute Chinese Academy of Tropical Agricultural Sciences Haikou 571101 China

**Keywords:** genome evolution, genomic variation, halophytes, salt stress, sesbania

## Abstract

Halophytes possess inherent stress resilience and diverse adaptations, making them valuable genetic reservoirs for crop breeding. The leguminous halophyte *Sesbania bispinosa* is a valuable forage crop that thrives in saline soils. To explore its salt tolerance, high‐quality genome assemblies is generated for the salt‐tolerant *S. bispinosa* accession SbTA02 and the salt‐sensitive accession SbSA44. Genomic analysis revealed that the genomic divergence between the two accessions primarily originates from their pericentromeric and centromeric regions, which contain the two largest inversions: a >27‐Mb inversion on chromosome 5 and a ≈49‐Mb inversion on chromosome 6. Population‐level analysis revealed that the 27‐Mb inversion is widespread in *S. bispinosa*, dividing the tested populations into inland and coastal groups. These groups have many genetic divergence regions (GDRs), with genetically isolated haplotypes in the middle section of chromosome 5, including the large inversion and centromeric regions. Genome‐wide association studies (GWAS) identified significant salt‐tolerance signals in the GDRs, pinpointing the anthocyanidin synthase gene *SbANS*. Natural variation in *SbANS* is associated with differences in salt tolerance between salt‐tolerant and salt‐sensitive *S. bispinosa* accessions. These findings provide insights into the genomic evolution of the *Sesbania* genus and shed light on how genomic variation shapes genome architecture, genetic divergence, and phenotypic differentiation.

## Graphical abstract

*Sesbania*, a leguminous halophyte, thrives in saline soils. Comparative genomics reveals key genomic variations—particularly chromosomal inversions—are identified as contributors to population differentiation and salt resilience. These findings advance the understanding of genomic variation driving evolution and phenotypic differentiation and offer valuable insights for crop improvement in saline environments.

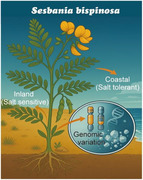

## 1 Introduction

Soil salinity is a pervasive problem affecting agricultural productivity worldwide. More than 10% of the world's croplands are affected by salinity, which reduces agricultural productivity and poses a major risk to global food security.^[^ ^1^ ^]^ Halophyte plants have exceptional adaptability to saline environments.^[^ ^2^ ^]^ Leveraging the knowledge and resources derived from halophytes could enable the development of new crop varieties that are better suited for saline agriculture, thereby enhancing agricultural productivity in challenging conditions.

Most studies on plant salt tolerance performed to date have focused on model plant species or domesticated crops grown under low salt concentrations, with relatively little attention given to halophytes.^[^ ^3^ ^]^ Studying halophyte plants holds substantial promise for enabling the development of salt‐tolerant crops, as these plants possess adaptations that allow them to flourish in highly saline environments.^[^ ^4^ ^]^ The genus *Sesbania* belongs to the Fabaceae family and comprises ≈60 species distributed worldwide in tropical and subtropical regions.^[^ ^5^ ^]^ These plants are widely cultivated as biofertilizers in agroforestry to improve soil fertility and serve as high‐protein (30–40% crude proteins) and multi‐nutrient forage crops.^[^ ^6^ ^]^ The allotetraploid plant *S. cannabina* (2*n* = 4*x* = 24) has exceptional tolerance to saline‐alkaline conditions and contributes significantly to the improvement of saline‐alkaline soil.^[^ ^7^ ^]^ The diploid plant *S. bispinosa* (2*n* = 2*x* = 12) is also salt tolerant and is commonly found in tropical and subtropical regions of China. It has small spines growing sparsely on its stem and petioles, and its leaves, flowers, pods, and seeds serve as sources of animal feed; moreover, mature *S. bispinosa* seeds are cooked and eaten by some Indian tribal sects.^[^ ^8^ ^]^ The resilience and economic benefits of *Sesbania* make it an ideal genus in which to study salinity tolerance and a potential resource for enhancing crop productivity and adaptability. To date, however, only the telomere‐to‐telomere genome of *S. cannabina* has been reported within the genus. Unexplored population resources and limited genomic information have hindered fundamental research and widespread utilization of *Sesbania* species.

In this study, we generated high‐quality genome sequences for two *S. bispinosa* accessions: the salt‐tolerant SbTA02 and the salt‐sensitive SbSA44. Comparative genomic analysis and CENH3 ChIP‐seq (chromatin immunoprecipitation sequencing of centromere‐specific histone H3) revealed a substantial divergence between the two genomes, which stems from inversions in peri‐/centromeric regions. Population analysis of 51 *S. bispinosa* accessions revealed that an ≈27‐Mb population‐level inversion on chromosome 5 divides genetically isolated haplotypes into two distinct groups. GWAS revealed that natural variation in the anthocyanin synthase gene *SbANS* is associated with differences in salt tolerance among members of the *S. bispinosa* population. Our comprehensive study illustrates how large and small mutations shape genomic divergence, population differentiation, and phenotypic evolution in *S. bispinosa*.

## 2 Result

### 2.1 Evaluation and Selection of Salt‐Tolerant *S. bispinosa* Accessions

**Figure 1:**
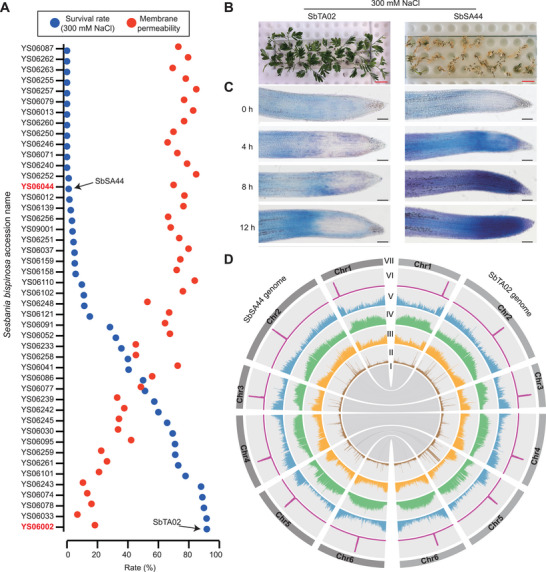
Evaluation of salt tolerance and genome assembly of *S. bispinosa*. A) Population‐level evaluation of salt tolerance in *S. bispinosa*. Membrane permeability and survival ratios under 300 mm NaCl were tested across *S. bispinosa* populations. The salt‐tolerant accession SbTA02 (YS06002) and salt‐sensitive accession SbSA44 (YS06044) are indicated by black arrows. B) Survival of SbTA02 and SbSA44 seedlings under salt stress. Scale bars, 2 cm. C) Cell viability of roots under 300 mm NaCl treatment at different timepoints (0–12 h) using trypan blue staining at room temperature. Scale bars, 100 µm. D) Genomic features of SbTA02 and SbSA44. I to VII represent, respectively, syntenic blocks; the contents of other‐type transposable elements (TEs), DNA‐type TEs, LTR retrotransposons, genes, and CENH3 ChIP‐seq signals; and chromosomes.

To systematically investigate the molecular regulatory mechanisms of salt tolerance and to develop new cultivars of *S. bispinosa*, we collected 51 natural populations of *S. bispinosa* from tropical and subtropical regions in China, 46 of which produced sufficient seeds for phenotypic analysis. We developed a system for evaluating the salt tolerance of *S. bispinosa* seedlings, whereby seedlings were grown until the first pinnate leaves fully expanded and then were treated with 300 mm NaCl (Figure S1A, Supporting Information). Seedling salt tolerance was assessed as survival rates and plant membrane permeability (**Figure** **1**A).

We observed a negative correlation between survival rates and plant membrane permeability: plants with lower survival rates tended to have higher membrane permeability. These results indicate that our evaluation system is reliable and stable. Fifteen accessions demonstrated a salt‐tolerant phenotype, with an average survival rate of >50%, whereas 14 accessions displayed a salt‐sensitive phenotype, with an average survival rate of <1.2%. The most salt‐tolerant accession was SbTA02 (accession no. YS06002), with a survival rate of 91.83 ± 2.25%; the salt‐sensitive accession was SbSA44 (accession no. YS06044), with a survival rate of 1.11 ± 1.57%. These accessions also exhibited other notable phenotypic differences and diversity, such as differences in plant morphology, small spine density, and biomass (Figure 1B; Figures S1B,C, and S2, Supporting Information).

We examined cell death of root cells in plants under salinity stress by performing trypan blue staining (Figure 1C). In the absence of NaCl treatment (at 0 h of treatment), all cells of both SbSA44 and SbTA02 plants survived. The root cell death rate in SbSA44 increased substantially beginning after 4 h of 300 mm NaCl treatment, reaching a peak at 12 h. By contrast, root cells in SbTA02 maintained a low cell death rate for up to 12 h of NaCl treatment. These accessions could be used as raw materials for scientific studies and could serve as foundations for selecting and breeding salt‐tolerant forage varieties.

### 2.2 Assembly and Annotation of two *S. bispinosa* Genomes

To explore genome evolution and salt‐tolerance mechanisms in *S. bispinosa*, we generated high‐quality genome assemblies of the highly salt‐tolerant accession SbTA02 and the salt‐sensitive accession SbSA44 (Figure 1D). Karyotyping using fluorescence in situ hybridization (FISH) revealed six pairs of chromosomes (2*n* = 2*x* = 12) in *S. bispinosa*, consistent with its diploid nature, as the base chromosome number is 6 (Figure S3A, Supporting Information). We constructed chromosome‐scale genome assemblies used PacBio HiFi reads (mean read length >15 kb), achieving ≈53‐fold coverage for SbSA44 and ≈57‐fold coverage for SbTA02, along with Hi‐C data at 205‐fold coverage for SbTA02 and 164‐fold coverage for SbSA44 (Figure S3B,C, and Table S1, Supporting Information).

The SbTA02 and SbSA44 genomes are 1090 and 1094 Mb in size, respectively (Table S2, Supporting Information). These assemblies comprise six chromosomes in 10 contigs for SbTA02 and 9 contigs for SbSA44. Only four genomic gaps in SbTA02 and three in SbSA44 were unresolved in the current assemblies. Furthermore, our assemblies featured 9 or 10 out of 12 complete chromosome ends, each exhibiting typical telomeric repeat units (Figure S3D, Supporting Information). All six large (>1 Mb) centromeres in both assemblies were successfully assembled; each is characterized by six prominent and central peaks in the middle chromosomal regions, as revealed by CENH3 ChIP‐seq (Figure 1D). The genome assemblies showed high assembly completeness, with a long terminal repeat (LTR) assembly index of 16.7–17.3 and base quality values of 51.8 for SbSA44 (single base accuracy 99.9993%) and 55.2 for SbTA02 (99.9997%) (Table S2, Supporting Information). The two genomes were predicted to contain 48 835 and 48 575 putative protein‐coding genes for SbTA02 and SbSA44, respectively. We assessed the completeness of the genomes using BUSCO, resulting in completeness scores of 99.2% for both assemblies (Table S2, Supporting Information). Both genome assemblies exhibit a high proportion of transposable elements, constituting ≈73% of their respective genomes. Notably, the largest family of these transposable elements is the LTR retrotransposons, which are predominantly localized to the central regions of the genomes (Figure 1D; Table S3, Supporting Information). This pattern is consistent with the distribution observed in most plant genomes with high transposable element contents.^[^ ^9^ ^]^

### 2.3 Genome Evolution and Variation Between *S. bispinosa* Genomes

**Figure 2:**
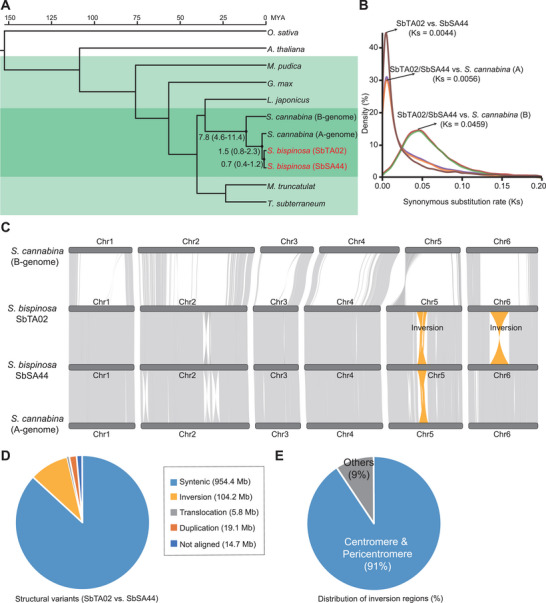
Genome evolution and comparisons between the two *S. bispinosa* genomes. A) Phylogenetic tree based on single‐copy protein‐coding genes. The light and dark green background represent Fabaceae and *Sesbania* species, respectively. B) Synonymous substitution rates among SbTA02, SbSA44, and the *S. cannabina* A‐ and B‐subgenomes. C) Macro‐synteny analysis of the two diploid *S. bispinosa* genomes and the A‐ and B‐subgenomes of allotetraploid *S. cannabina*. The two large inversions on chromosomes 5 and 6 are highlighted in orange. D) Analysis of structural variants between SbTA02 and SbSA44. E) Statistics for the distribution of inversion regions.

Extensive research has shown that variation is a major driving force behind genome evolution and trait determination.^[^ ^10^ ^]^ Our phylogenetic analysis suggested that the two *S. bispinosa* genomes diverged from one another ≈0.7 million years ago (Mya) (**Figure** **2**A) and share a most recent common ancestor with the A‐subgenome of *S. cannabina* ≈1.5 Mya. By contrast, the B‐subgenome of *S. cannabina* shows a more distant evolutionary relationship with *S. bispinosa*, with a divergence time estimated at 7.8 Mya. Synonymous mutation analysis corroborated these evolutionary timelines (Figure 2B), reinforcing the hypothesis that *S. bispinosa* is more closely related to the A‐subgenome than the B‐subgenome of *S. cannabina* and likely shares a recent common ancestor with the former.

Genomic synteny analysis revealed that the two largest inversions between the two *S. bispinosa* accessions were a >27‐Mb inversion located on chromosomes 5 (at positions 74.79–101.93 Mb) and a ≈49‐Mb inversion located on chromosome 6 (at positions 53.86–102.58 Mb), as confirmed by Hi‐C data (Figure 2C; Figure S4, Supporting Information). Moreover, the *S. bispinosa* genome exhibits a high degree of collinear blocks with the A‐subgenome of the allotetraploid *S. cannabina*,^[^ ^7^ ^]^ whereas there is substantial divergence with the B‐subgenome, particularly in the middle chromosomal regions (Figure 2C). Comparative analysis indicated that the genomes of *S. bispinosa* SbTA02 and SbSA44 contain 954.4 Mb of syntenic sequences (87.2% of genome sequences) but a significant proportion of variable sequences (12.8% of genome sequences), which mainly arise from ≈104.2 Mb of inversions (Figure 2D; Figure S4A, Supporting Information). Notably, 91% of these inversion regions overlap with centromeric or pericentromeric regions (Figure 2E). Collectively, these findings indicate that the centromeric and pericentromeric regions serve as genomic divergence hotspots between the two *S. bispinosa* genomes, as they are characterized by substantial structural variations.

### 2.4 Comparison of Centromeric Sequences and Structures Reveals Genomic Divergence

**Figure 3:**
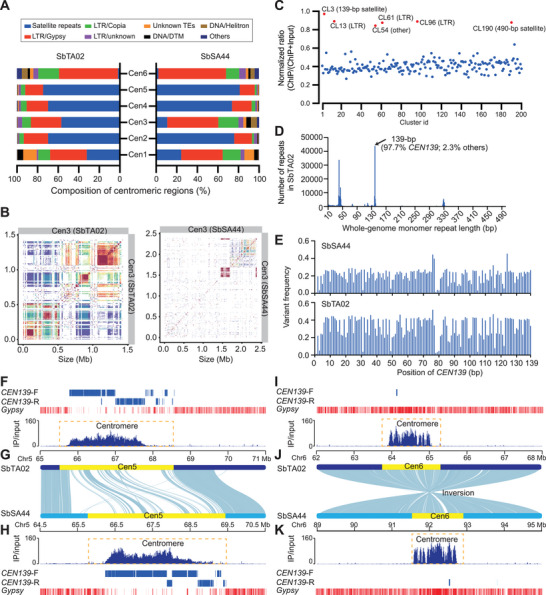
Centromeric sequences and structures of the two *S. bispinosa* genomes. A) Component analysis of the six centromeres of the SbTA02 and SbSA44 genomes. Cen1 to Cen6 represent six centromeres from different chromosomes. B) Identity heatmap of the centromere sequences from chromosome 3. C) CENH3 ChIP‐seq reads mapped against the first 200 most abundant repeat clusters of the SbSA44 genome identified by RepeatExplorer. The major centromeric sequences (normalized ratio ≥ 0.8) identified by CENH3 ChIP‐seq are highlighted with red dots. D) Length distribution of the monomer repeat across the whole SbTA02 genome identified using TRASH tools. E) Sequence variants of the *CEN139* family across different base positions in SbTA02 and SbSA44. The x‐axis and y‐axis represent base position and variant frequency, respectively. F–H) Distribution patterns of major centromeric repeats (F,H) and sequence comparisons of Cen5 with abundant *CEN139* G). *CEN139‐F* and *CEN139‐R* represent the forward and reverse sequences of *CEN139*, respectively. The blue peaks represent the CENH3 ChIP‐seq signals. I–K) Distribution patterns of major centromeric repeats I,K) and sequence comparisons of Cen6 without remnants of *CEN139* J).

Centromeres in plants are characterized by satellite repeats and transposable elements.^[^ ^11^ ^]^ To perform a detailed examination of centromere structure in *S. bispinosa*, we generated a customized *Sesbania*‐specific anti‐CENH3 antibody and performed CENH3 ChIP‐seq, which allowed us to successfully pinpoint all centromere regions in the SbTA02 and SbSA44 genomes (Figure S5 and Table S4, Supporting Information). Component analysis revealed that, except for the centromere in chromosome 6 (Cen6), the five other centromeres mainly comprise LTR retrotransposon fragments and centromeric satellite repeats (**Figure** **3**A). Notably, unlike most tandem repeat families in plants, such as the satellite repeat sequences in *Arabidopsis* (*Arabidopsis thaliana*),^[^ ^11b^ ^]^ *Erianthus rufipilus* (a diploid *Saccharum*),^[^ ^12^ ^]^ and soybean (*Glycine max*) centromeres,^[^ ^13^ ^]^ Cen6 in *S. bispinosa* contains remnants of centromeric satellite repeats but is predominantly composed of LTR retrotransposons. This is similar to what has been observed in diploid cotton (*Gossypium raimondii*),^[^ ^14^ ^]^ potato (*Solanum tuberosum*),^[^ ^15^ ^]^ and rye (*Secale cereale*).^[^ ^16^ ^]^ Identity heatmap analysis showed that the centromeric repeats in Cen6 exhibited little sequence similarity, but Cen1–Cen5 displayed high sequence similarity and tended to homogenization, with abundant centromeric satellite repeats (Figure S6, Supporting Information). Of these centromeres, Cen3 showed substantial differences between the SbTA02 and SbSA44 genomes, with a notable reduction in satellite repeats and expansion in LTR retrotransposon fragments for SbSA44, distinguishing its structure from that of Cen3 in SbTA02 (Figure 3B). Collectively, these results indicate that satellite homogenization and retrotransposon‐driven diversification are two major models driving centromere evolution and divergence for the two genomes.

To identify functional centromeres that bind to CENH3 nucleosomes, we mapped the CENH3 ChIP‐seq reads from SbSA44 onto genome‐wide repeat clusters using RepeatExplorer to confirm the association of major clusters with functional centromeres (Figure 3C). Analysis of ChIP‐seq reads demonstrated that the six repeat elements (CL3, CL13, CL54, CL61, CL96, and CL190) in SbSA44 were among the clusters that showed the highest levels of association in the immunoprecipitated fraction (Figure 3C). Of these major clusters, we identified two satellite repeats (139 and 490 bp) and three LTR retrotransposon fragments, indicating that CENH3 binds satellite repeats and LTR retrotransposon fragments. Analysis using the Tandem Repeat Annotation and Structural Hierarchy (TRASH) tool revealed that the actual unit length of the satellite repeats matched that of *CEN139*, with a unit length of 139 bp (Figure 3D). To explore the characteristics of the *CEN139* family, we derived the consensus sequences for *CEN139* and examined the base variation within the respective consensus sequence. Our analysis revealed that the *CEN139* family in the SbTA02 genome exhibited more variation compared to the *CEN139* in the SbTA44 genome, indicating that a more rapid evolutionary process has occurred in the SbTA02 genome (Figure 3E). Additionally, the core centromeric regions exhibited sequence divergence due to the distinct abundance and arrangement of *CEN139* and LTR retrotransposon fragments, contrasting with the surrounding areas where syntenic blocks were more prevalent (Figure 3F–K; Figure S7, Supporting Information). These findings suggest that, despite the recent divergence of the two genomes (≈0.7 Mya), significant variations in centromeric regions have accumulated, potentially driving their speciation.

### 2.5 Single‐Nucleotide Polymorphism Analysis Reveals Geographic Population Differentiation

The genetic basis of key agronomic traits has been extensively studied in natural populations of many crops, such as rice (*Oryza sativa*),^[^ ^17^ ^]^ cotton,^[^ ^18^ ^]^ and soybean,^[^ ^19^ ^]^ but similar studies have not been conducted in *Sesbania* species. We generated a total of ≈1145 Gb of 150‐bp paired‐end reads for 51 diploid *S. bispinosa* accessions, with an average coverage depth of ≈20.6× for each accession (Table S5, Supporting Information). On average, 94.28% of the reads for each accession were successfully aligned with the SbTA02 reference genome for the identification of single‐nucleotide polymorphisms (SNPs). We identified 2 918 655 high‐quality SNPs (minor allele frequency ≥0.03 and missing rate ≤0.1) in the *S. bispinosa* accessions, an average of 2.7 SNPs per kilobase.

**Figure 4:**
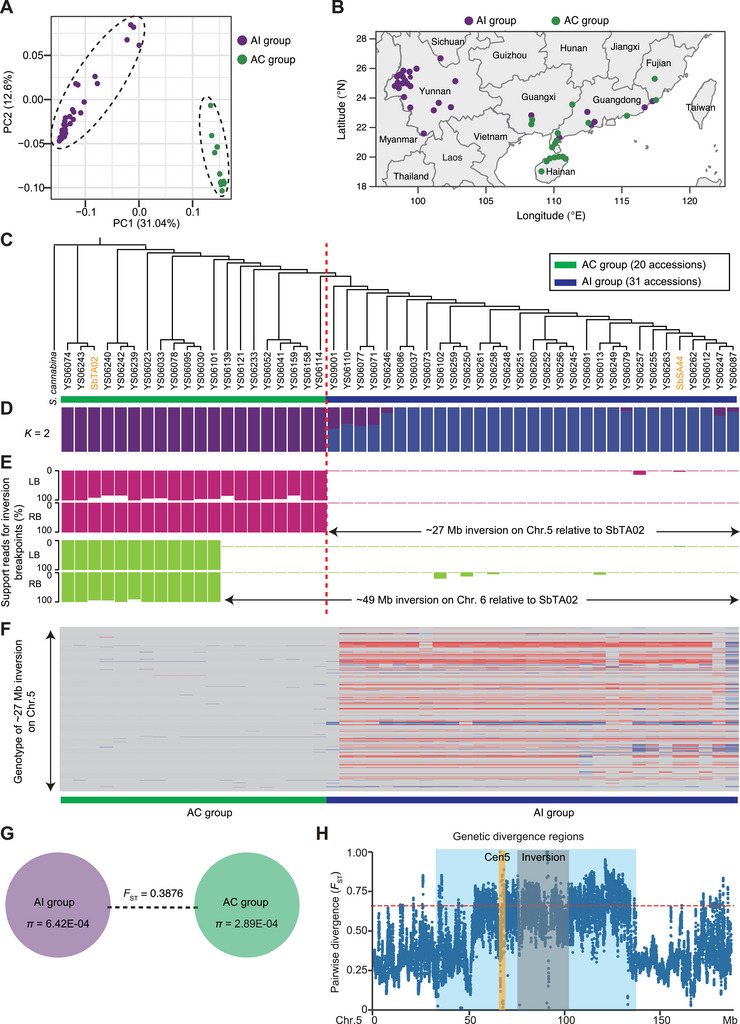
Population analysis of *S. bispinosa* reveals highly divergent genetic regions. A) Principal component analysis (PCA) of the first two components supports the division of the accessions into two groups. AI and AC indicate accessions from inland and coastal regions, respectively. B) Geographic distribution of *S. bispinosa* accessions. C) Phylogenetic analysis based on whole‐genome SNPs, with *S. cannabina* as the outgroup. D) Population structure analysis with a kinship coefficient of 2. E) Genetic effects of the large inversions on chromosome 5 and 6. For each inversion breakpoint, the ratio of supporting reads for the inversion breakpoints was calculated by dividing the number of reads at the corresponding breakpoints in SbTA02 by the total number of reads at the breakpoints in both genomes (SbTA02 and SbSA44). F) Genotype analysis of the ≈27‐Mb inversion on chromosome 5. Gray, red, and blue represent the same allele as that of SbTA02, the alternative allele (same as that of SbSA44), and heterozygous alleles, respectively. G) Pairwise fixation statistic (*F*_ST_) and diversity (π) for the AI and AC groups. H) Characterization of the highly genetically divergent regions (GDRs) located from 34.0 to 136.7 Mb in the middle of chromosome 5, including Cen5 and a ≈27‐Mb large inversion. The vertical light blue shadow indicates genetic divergence regions.

Principal component analysis (PCA) based on genome‐wide SNPs classified all samples into two distinct groups (**Figure** **4**A). We defined the accessions from inland and coastal regions as AI and AC, respectively. The AC group is found distributed in coastal cities, whereas the AI group is primarily distributed in inland regions (Figure 4B). Phylogenetic analysis and population structure analysis further confirmed the existence of two distinct groups (Figure 4C,D). Thus, our data demonstrate that *S. bispinosa* exhibits pronounced geographical distribution patterns, indicating that environmental and ecological factors likely have significant influences on its adaptation and spatial spread.

### 2.6 Inversions Influenced the Formation of Natural Haplotypes

Inversions have a strong effect on natural haplotype by drastically suppressing recombination, thereby reducing genetic exchange.^[^ ^20^ ^]^ We investigated whether the *S. bispinosa* accessions exhibited population‐wide inversions and estimated their effects on natural haplotypes. We analyzed the two largest inversions between the SbTA02 and SbSA44 genomes—the ≈27‐Mb inversion on chromosome 5 and the ≈49‐Mb inversion on chromosome 6—across the *S. bispinosa* population (Figure 4E). Specifically, we aligned the resequencing reads against the SbTA02 genome and calculated the ratio of supporting reads for the left and right inversion breakpoints.

Notably, sequencing reads from the AC group matched the ≈27‐Mb inversion breakpoint on chromosome 5 in the SbTA02 genome, but sequencing reads from the AI group did not align to the corresponding inversion breakpoints (Figure 4E), suggesting that the 27‐Mb inversion might divide the population into two clades. These two clades (based on breakpoint genotyping of the ∼27‐Mb inversion) match the grouping based on PCA, phylogenetic analysis, and population structure (Figure 4F). By contrast, breakpoint genotyping of the ≈49‐Mb inversion on chromosome 6 revealed that only 12 of 20 accessions from the AC group exhibited the inversion breakpoints found in the SbTA02 genome (Figure 4E). These findings suggest a potential link between the 27‐Mb inversion and population differentiation, potentially leading to genetically isolated haplotypes in the *S. bispinosa* population.

Comparisons of pairwise fixation statistic (*F*_ST_) values for the AI and AC groups revealed a high *F*_ST_ value of 0.3876, indicative of noteworthy genetic differentiation (Figure 4G). The average nucleotide diversity (*π*) was lower (2.89 × 10^−4^) for the AC group than for the AI group (6.42 × 10^−4^). These results suggest that *S. bispinosa* may have spread from the southwestern inland region to the eastern coastal regions. The top 5% of *F*_ST_ values were higher in the inversion region on chromosome 5 than in the other regions, and this effect extended into the peri‐/centromeric regions, which comprise the genetic divergence regions (GDRs) located at 34.0–136.7 Mb on chromosome 5 (Figure 4H; Table S6, Supporting Information). By contrast, no substantial genetic divergence was observed in the ≈49‐Mb inversion on chromosome 6 (Figure S8, Supporting Information). These highly divergent genomic regions have driven the differentiation of this population, which might have led to phenotypic diversity within the *S. bispinosa* population.

### 2.7 GWAS Shows that Salt Tolerance is Associated with an Anthocyanidin Synthase Gene

**Figure 5:**
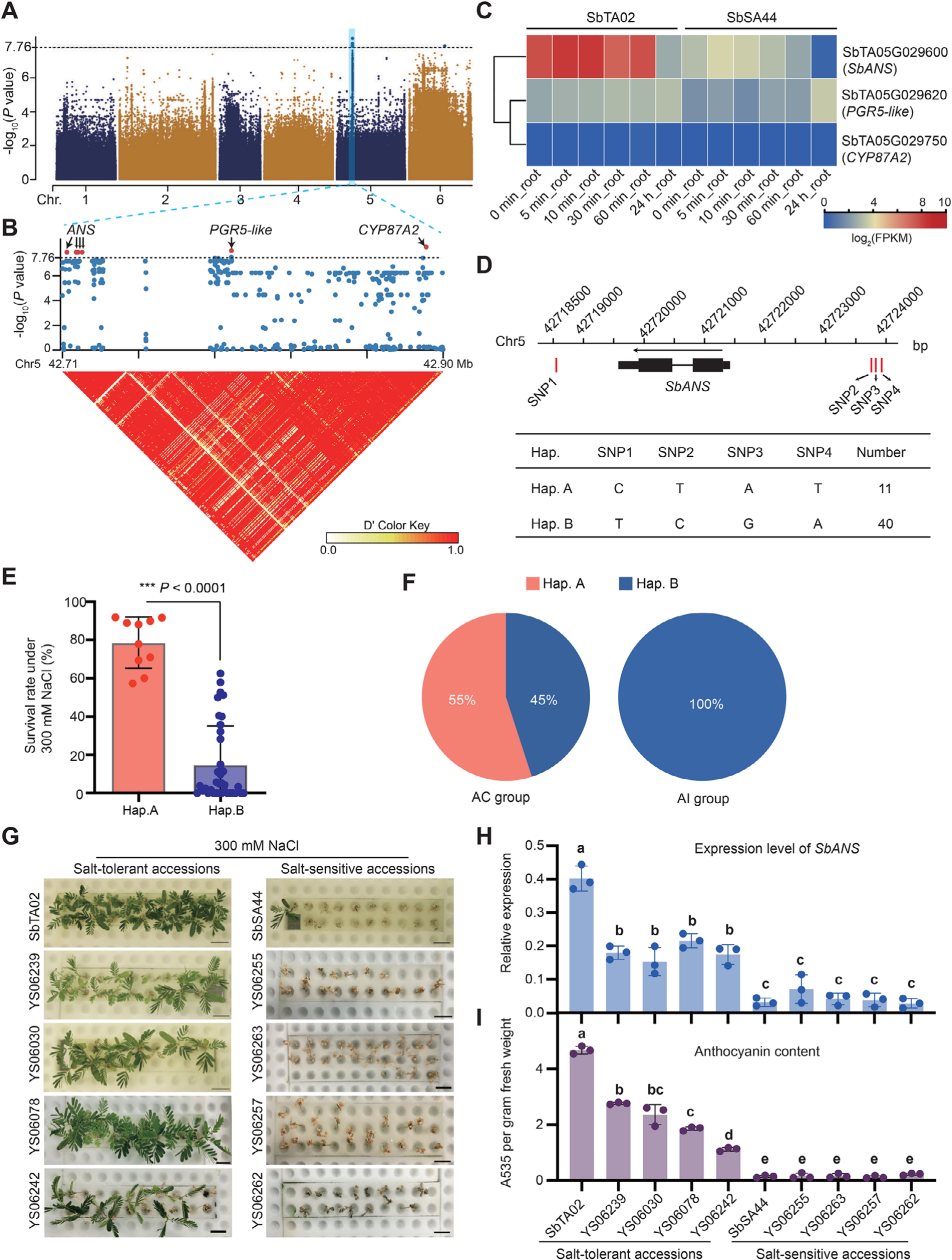
Identification of a genetic locus that contributes to the differential salt tolerance in the *S. bispinosa* population. A) GWAS examining salt tolerance in *S. bispinosa*. The black dashed line and light blue highlighting indicate the threshold (–log_10_(*p*‐value) ≥ 7.76) for GWAS signals and the significant locus, respectively. B) Local Manhattan plot (top) and LD heat map (bottom) surrounding the peak on chromosome 5. The significant SNPs with neighboring genes are indicated by black arrows. C) Expression profiles of candidate genes with significant SNPs in SbTA02 and SbSA44 roots at different time points during NaCl treatment (0 min to 24 h). D) Gene structure (top) and haplotype analysis (bottom) of the anthocyanidin synthase gene *SbANS*. Black rectangles and line indicate exons and introns, respectively. The four significant SNPs (SNP1 to SNP4) are indicated by red lines. E) Comparison of survival rates under 300 mm NaCl treatment between haplotypes A and B in the GWAS population. Data are presented as the mean ± s.d. ^***^ *p* < 0.001 (two‐tailed Student's *t*‐test). F) Distribution of allele frequencies and geographical distribution of significant SNPs in the GWAS population. The salt‐tolerant (Hap. A) and salt‐sensitive (Hap. B) alleles are shown in orange and blue, respectively. G) Phenotypic observation of five selected salt‐tolerant accessions (left) and five salt‐sensitive accessions (right). Scale bars, 2 cm. H,I) *SbANS* expression level H) and anthocyanin content I) in roots without NaCl treatment from salt‐tolerant and salt‐sensitive accessions. *Actin* was used as the internal control for qRT‐PCR. Data are presented as the mean ± s.d. *n* = 3 independent experiments for each sample. A one‐way ANOVA using Tukey's multiple comparisons test at a 99.9% confidence interval was performed to identify significant differences between samples (indicated by different letters above bars).

Uncovering the genes responsible for salt tolerance is crucial for enhancing plant adaptation to saline environments and making better use of saline soils.^[^ ^21^ ^]^ Although *Sesbania* species are recognized for their excellent salt tolerance, its underlying mechanisms and the associated genes have yet to be identified. We performed a GWAS for salt tolerance in *S. bispinosa*, as measured by the survival rate under 300 mm NaCl treatment (**Figure** **5**A). We identified six strong association signals on chromosome 5 with a—log_10_(*p*‐value) ≥ 7.76. The six significant signals were localized in three nearby genes encoding proteins including anthocyanidin synthase (ANS; gene locus: SbTA05G029600), PGR5‐like protein 1A (PGR5‐like; gene locus: SbTA05G029620), and Cytochrome p450 (CYP87A2; gene locus: SbTA05G029750) (Figure 5B, upper panel). These signals comprise a linkage block located at 42.71–42.90 Mb on chromosome 5 (Figure 5B, lower panel). Notably, the linkage block overlapped with the GDRs mentioned above.

All six significant SNPs were in non‐coding sequences with no effects on protein sequences, suggesting the SNPs might affect the expression levels of these genes. Additionally, 4 out of 6 significant SNPs were located in the upstream or downstream regions of *SbANS*, an ortholog of the *Medicago truncatula* gene encoding ANS/LDOX, an enzyme involved in converting leucoanthocyanidin to anthocyanidins^[^ ^22^ ^]^ (Figure S9, Supporting Information).

To pinpoint the key salt‐tolerance gene, we conducted RNA‐seq of root tissues from the salt‐sensitive accession SbSA44 and the salt‐tolerant accession SbTA02 at various time points of NaCl treatment, ranging from 0 min to 24 h (Table S7, Supporting Information). We identified 2067, 1857, 1785, 2020, 1677, and 1509 differentially expressed genes (DEGs) between the two samples after 0, 5, 10, 30, 60, and 24 h of salt treatment, respectively (fold change ≥ 2 and *p*‐value ≤ 0.05) (Tables S8–S13, Supporting Information). Gene Ontology (GO) enrichment analysis of the DEGs showed that both the proanthocyanidin biosynthetic process and flavonoid metabolic process were common significantly enriched biological process terms (Figures S10 and S11, Supporting Information). Oxidoreductase activity was also significantly enriched, indicating that mitochondrial electron transport chain activity may play an active role in salt stress adaptation.

Notably, only the *SbANS* gene exhibited significantly higher expression in SbTA02 compared to SbSA44 during NaCl treatment, whereas the two other genes were expressed at low levels, with no differential expression between SbTA02 and SbSA44 (Figure 5C). We identified two haplotypes (Hap. A and Hap. B) based on four significant SNPs near the *SbANS* gene (Figure 5D). Accessions with haplotype A demonstrated significantly higher salt tolerance than those with haplotype B (Figure 5E). All members of the AI group (primarily from inland regions) carried the salt‐sensitive haplotype B, whereas 55% of the AC group (from coastal regions) carried the salt‐tolerant haplotype A and 45% carried the salt‐sensitive haplotype B (Figure 5F). These results suggest that salt tolerance has changed substantially during the migration of *S. bispinosa* from southwestern inland to coastal regions.

To investigate the role of *SbANS* in salt tolerance in *S. bispinosa*, we selected five salt‐tolerant and five salt‐sensitive accessions for detailed analysis (Figure 5G). qRT‐PCR analysis showed that *SbANS* in the salt‐tolerant accessions maintained significantly higher expression levels in the absence of NaCl treatment, whereas *SbANS* in the salt‐sensitive accessions exhibited low expression levels (Figure 5H; Figure S12, Supporting Information). The *SbANS* expression analysis across population‐level accessions suggests that accessions with higher *SbANS* expression levels are predominantly from the AC group, while those from the AI group tend to show lower expression levels (Figure S13, Supporting Information). This expression pattern corresponded with anthocyanin contents: the highly salt‐tolerant accessions showed significant anthocyanin accumulation, whereas the salt‐sensitive accessions had lower anthocyanin contents (Figure 5I). We also conducted complementary functional validation by overexpressing *SbANS* in soybean to assess its conserved role in salt tolerance (Figure S14, Supporting Information). Phenotypic observations and quantitative growth parameter measurements revealed significant differences between transgenic and control plants under salt stress, demonstrating that elevated expression of *SbANS* could enhance salt tolerance in soybeans. Collectively, these results suggest that natural variation in the *SbANS* gene is associated with the differentiation in salt tolerance between the two groups of *S. bispinosa* accessions.

## 3 Conclusion

In this study, we generated the first two high‐quality genomic resources for *S. bispinosa*, each with a genome size of ≈1 Gb with all centromeres resolved. Through comparative genomics, we investigated the effect of genomic variation on sequence divergence and population differentiation among *S. bispinosa* accessions. We determined that natural variation in the anthocyanidin synthase gene *SbANS* confers differences in salt tolerance among *S. bispinosa* populations. Our findings provide valuable genomic resources for comparative studies in *Sesbania* and identify key loci that could be targeted for the genetic improvement of salt‐tolerance traits in other plants.

There is a growing appreciation that genomic variations, particularly inversions and translocations, play crucial roles in plant adaptation and speciation, yet their biological implications are often underexplored.^[^ ^23^ ^]^ Comparative genomic studies have identified many new paracentric inversion polymorphisms linked to plant adaptation and trait formation. Large inversions suppress recombination by reducing crossing over, as exemplified by a 5.8‐Mb inversion associated with carotenoid content in potato tubers.^[^ ^10b^ ^]^ Researchers have identified strong associations between paracentric inversions and fecundity in *Arabidopsis* under drought stress, with linkage disequilibrium observed between the inverted region and early flowering, suggesting that these genetic elements enhance the maintenance and spread of the inversion.^[^ ^24^ ^]^ The large inversions on chromosome A08 of allotetraploid cotton in a large germplasm diversity panel enhance the capacity of cotton cultivars to adapt to ecological environments.^[^ ^25^ ^]^

Here we determined that a paracentric inversion on chromosome 5 is prevalent among natural *S. bispinosa* accessions. Intriguingly, like the A‐ and B‐subgenomes of allotetraploid *S. cannabina*, the large inversions in the A‐ and B‐subgenomes of *S. bispinosa* are also located near the terminal region of chromosome 5,^[^ ^7^ ^]^ perhaps underscoring the tendency for substantial genomic variations on chromosome 5. These genomic variations on chromosome 5 likely played a pivotal role in facilitating species differentiation within the *Sesbania* genus and may confer a distinct selective advantage to various species in adapting to their respective local environments. However, identifying the specific traits that are affected by this large inversion remains quite challenging. In the future, the construction of a cross‐population between accessions with and without the inversion, followed by genetic analysis and phenotypic characterization, should help unravel the influence of this inversion on various traits.

Our findings indicate that natural variations in the regulatory regions of the anthocyanin synthesis gene *SbANS* are linked to the differentiation of salt tolerance traits in *S. bispinosa*. The ectopic expression of the *LDOX/ANS* gene from the desert halophyte *Reaumuria trigyna* resulted in flavonoid accumulation and improved salt tolerance in *Arabidopsis*.^[^ ^26^ ^]^ Notably, we demonstrated here that the natural variations in the regulatory regions of the *ANS* gene are key factors influencing plant salt tolerance. This finding highlights the importance of natural variation in enhancing resistance traits, whereas previous studies have focused on protein functions instead of exploring the significance of natural variation in regulatory regions within natural populations.

Furthermore, our data indicate that long‐term natural selection under environmental stress has endowed wild *S. bispinosa* with enhanced salt tolerance. Plants synthesize anthocyanins to mitigate ionic and osmotic damage and scavenge reactive oxygen species in response to salinity^[^ ^4^, ^27^ ^]^; by contrast, the biosynthesis of anthocyanins in salt‐tolerant *S. bispinosa* is constitutive rather than inducible (Figure S12, Supporting Information). This contrasts with species such as *R. trigyna* ^[^ ^26^ ^]^ and carrot (*Daucus carota*),^[^ ^28^ ^]^ whose flavonoid pathway‐related genes are strongly upregulated under salt stress. The constitutive biosynthesis of anthocyanins in salt‐tolerant *S. bispinosa* likely represents an adaptive mechanism shaped by evolutionary pressures, enabling it to thrive in saline coastal environments where constant protection is required.

## 4 Experimental Section

### Plant Materials

The seeds of *S. bispinosa*, provided by the Chinese Academy of Tropical Agricultural Sciences, were collected from tropical and subtropical regions in China. Plants were self‐fertilized and maintained in the greenhouse. DNA samples were obtained from fresh young leaves of a single mature SbTA02 (accession number YS06002) and SbSA44 (accession no. YS06044) plant and subjected to genome sequencing and assembly.

### Evaluating Salt Tolerance in S. Bispinosa

To evaluate salt tolerance, the seeds were polished for ≈10 s using a grain polisher (Pearlest Grain Polisher) and germinated for ≈4 days on moistened filter paper. The germinated seedlings were transferred to sponges and grown hydroponically in 1/2 Hoagland nutrient solution (Coolaber, NSP1020) under controlled conditions in an illuminated incubator (14 h of light/10 h of darkness, 60% relative humidity, and a light intensity of 100 µmol m^−^^2^ s^−1^). When the plants reached the first compound leaf stage, they were treated with 300 mm NaCl for 2 days. After salt treatment, the plants were returned to 1/2 Hoagland nutrient solution without NaCl treatment for recovery, and their survival rates were assessed 5 days later. Each experiment used 20 seedlings.

### Cell Viability Assay Under NaCl Treatment

The cell viability assay was conducted using trypan blue staining. Briefly, root tissues were treated with 300 mm NaCl and sampled at various timepoints (0, 4, 8, and 12 h) for staining with 0.4% trypan blue (Sigma Aldrich, St. Louis, MO, USA). Stained cells were examined under a light microscope, and images were captured using a CCD camera.

### Genome Sequencing and Assembly

PacBio HiFi reads with a mean read length of 15 043 bp for SbTA02 and 17 132 bp for SbSA44 were generated using the PacBio Revio system, resulting in 62 920 785 361 bp of PacBio HiFi reads for SbTA02 and 58 630 947 683 bp for SbSA44. In the Hi‐C experiments, cross‐linked chromatin was digested into fragments using the restriction enzyme MobI, and a Hi‐C library was constructed following the standard protocol. A total of 225 051 769 800 bp of sequences for SbTA02 and 180 745 731 300 bp for SbSA44 were generated in 150 bp paired‐end mode on the DNBSEQ‐T7 sequencing platform from MGI Technology. Additionally, 79 320 061 500 bp of MGI reads for SbTA02 and 83 559 890 400 bp for SbSA44 were sequenced at the whole genome level.

CCS reads generated by the PacBio platform were used for preliminary genome assembly using Hifiasm (version 0.19.8‐r603) with default parameters.^[^ ^29^ ^]^ The assembly process involved three major steps: first, a comprehensive comparison was performed to correct sequencing errors; second, the corrected reads were stacked to construct a contact graph; and third, large contigs were generated from the overlap graph. Subsequently, the Hi‐C data underwent preliminary filtering using SOAPnuke (Version 2.1.7) with the parameters “‐l 20 ‐q 0.5 ‐n 0.1.”^[^ ^30^ ^]^ The filtered reads were aligned to the preliminary assembly using BWA software (Version 0.7.17‐r1188)^[^ ^31^ ^]^ following the pipeline outlined at https://github.com/ArimaGenomics/mapping_pipeline. The order and orientation of each cluster and scaffold were confirmed and anchored to pseudo‐chromosomes using YaHS (Version 1.2a.1).^[^ ^32^ ^]^ Manual corrections were performed and the final chromosome‐level reference genome was generated with juicer_tools (Version 1.9.9) and Juicer (Version 1.1)^[^ ^33^ ^]^; the detailed procedures are available at https://github.com/c‐zhou/yahs. The quality of the assembled reference genome was assessed using BUSCO (Version 5.3.2)^[^ ^34^ ^]^ and Merqury (Version 1.3).^[^ ^35^ ^]^ Finally, Hi‐C heatmaps were generated using HiC‐Pro (Version 3.1.0)^[^ ^36^ ^]^ and HiCPlotter (Version 0.6.02).^[^ ^37^ ^]^

### Annotation of Transposable Elements and Genes

For genome‐wide annotation of transposable elements, the EDTA pipeline was used, with parameters set to “–step all –sensitive 1 –anno 1.”^[^ ^38^ ^]^ Additionally, QuarTeT^[^ ^39^ ^]^ was used to predict centromeres and telomeres, and LTR_retriever^[^ ^40^ ^]^ was used to calculate the LTR Assembly Index to assess genome quality. Gene annotation was performed using a previously described pipeline for *de novo* predictions of protein‐coding genes.^[^ ^7^ ^]^ Transcriptome data were assembled for gene prediction (Table S7, Supporting Information). Homology predictions were performed using protein sequences from closely related species with GeMoMa,^[^ ^41^ ^]^ and MAKER^[^ ^42^ ^]^ was used to integrate and refine the resulting predictions.

### Detection of Genomic Structural Variations

SbTA02 was used as the reference genome and SbSA44 as the query genome to identify structural variations. Specifically, genome alignment was performed using MUMmer (version 4.0.0)^[^ ^43^ ^]^ with default parameters and filtered using the criteria minimum alignment length ≥1000 bp and minimum alignment identity ≥95, allowing for rearrangements. The filtered aligned pairs served as input for SyRI (version 1.4)^[^ ^44^ ^]^ with default parameters. The genomic structural variations from the SyRI results were categorized as structural annotations (syntenic regions, inversions, translocations, duplications, non‐aligned regions) and sequence annotations (SNPs, insertions, deletions, copy gains, copy losses, and highly divergent regions).

### Centromere Identification and Identity Heatmap Analysis

A rabbit polyclonal anti‐CENH3 antibody specific to *Sesbania* was developed using the peptide ARVKHAPAGPHRRN, which was derived from the N‐terminal region of CENH3 in *S. bispinosa*. CENH3 ChIP‐seq experiments were performed as described previously^[^ ^45^ ^]^ using the newly developed *Sesbania*‐specific anti‐CENH3 antibody. Approximately 20 g samples of young leaf tissue were collected from SbTA02 and SbSA44. The enriched and input DNA samples were sequenced on the HiSeq 2000 platform, generating paired‐end 150 bp sequencing reads. The reads were aligned to the corresponding genome assembly using Bowtie 2 (v.2.2.5) (Langmead and Salzberg, 2012). The read depth at each position across the chromosomes was calculated with the BEDtools package, normalizing each position based on the total number of reads for the corresponding sample. The immunoprecipitation‐to‐input DNA ratio was then computed, and mean fold enrichment was determined in 10‐kb non‐overlapping windows across each chromosome using custom scripts. To delineate the centromere domain, SICER2 (v.1.0.2)^[^ ^46^ ^]^ was employed to identify the significant CENH3 peaks, setting the parameters to (–window_size 200–false_discovery_rate 0.01–gap_size 400).

RepeatExplorer^[^ ^47^ ^]^ was also used to identify and characterize the functional centromeric monomer repeats based on CENH3 ChIP‐seq data. RepeatExplorer utilities including clustering, interlaced, and ChIP‐seq mapper were employed using the Galaxy‐based web interface (https://repeatexplorer‐elixir.cerit‐sc.cz/galaxy/). Tandem repeats were identified and mapped using TRASH^[^ ^48^ ^]^ based on the genome sequences of SbTA02 and SbSA44. This approach helped anchor centromeric tandem repeats and their higher‐order structures. To visualize centromeric structures, colored identity heatmaps were constructed using StainedGlass with a window size of 2000 bp.^[^ ^49^ ^]^

### Population Genomics Analysis

DNA was extracted from members of the *S. bispinosa* population and used to construct libraries using the MGISEQ sequencing platform. The raw sequencing data were filtered using SOAPnuke (Version 2.1.7)^[^ ^30^ ^]^ and mapped to the SbTA02 genome using BWA software (Version 0.7.17‐r1188).^[^ ^31^ ^]^ After removing of duplicate reads with Picard (Version 2.27.5) (https://broadinstitute.github.io/picard/), the filtered reads were used to identify SNPs and insertions/deletions (InDels) using GATK (Version 4.3.0.0)^[^ ^50^ ^]^ with the criteria “QD < 2.0 || MQ < 40.0 || FS > 60.0 || SOR > 3.0 || MQRankSum <‐12.5 || ReadPosRankSum < ‐8.0” and “QD < 2.0 || FS > 200.0 || SOR > 10.0 || MQRankSum < ‐12.5 || ReadPosRankSum < ‐8.0.” The high‐quality SNPs were further filtered using PLINK (v1.90b4.6)^[^ ^51^ ^]^ with a minor allele frequency (MAF) ≥ 0.03 and a missing rate ≤ 0.1.

For phylogenetic analysis, the filtered SNPs were analyzed using the maximum likelihood method with IQ‐TREE (Version 2.2.2.6),^[^ ^52^ ^]^ which automatically selects the best model and employs ultra‐fast bootstrap to perform 1000 bootstrap replicates. This ultimately led to the inference of the phylogenetic tree, with the parameters “‐keep‐ident –seqtype DNA ‐m MFP+ASC ‐B 1000 ‐bnni.” The phylogenetic tree was visualized and enhanced using the online tool iTOL (https://itol.embl.de/).

For principal component analysis (PCA), the SNPs were filtered (MAF ≥ 0.03 and a missing rate ≤ 0.1), and PCA was performed using PLINK (Version v1.90b4.6).^[^ ^51^ ^]^ Nucleotide diversity (π) and *F*_ST_ were calculated and analyzed using VCFtools (Version 0.1.16), with a window and step size set to 100 000 and 10 000 bp, respectively. Additionally, population structure analysis was performed using ADMIXTURE (Version 1.3.0),^[^ ^53^ ^]^ with further LD filtering via PLINK using the parameter “–indep‐pairwise 50 10 0.1.”

### Genotyping of Inversion Breakpoints

Genotyping of inversion breakpoints was performed as described previously.^[^ ^20^, ^25^ ^]^ In brief, the filtered resequencing reads from *S. bispinosa* populations were mapped to the SbTA02 and SbSA44 genomes using BWA software (Version 0.7.17‐r1188).^[^ ^31^ ^]^ For each inversion breakpoint, the ratio of reads at the breakpoint was calculated by dividing the number of reads at the corresponding breakpoints in SbTA02 by the total number of reads at the breakpoints in both genomes (SbTA02 and SbSA44). This analysis revealed two distinct ratio patterns, allowing us to differentiate between the SbSA44 inversion haplotype and the SbTA02 inversion haplotype using the ratio ranges of 0 to 20% and 80% to 100%.

### Genome‐Wide Association Study (GWAS)

GWAS for salt tolerance was performed using high‐quality SNPs (MAF ≥ 0.03 and a missing rate ≤ 0.1) in 46 wild diploid *S. bispinosa* accessions using the general linear model in Tassel (version 5.2.40)^[^ ^54^ ^]^ with the PCA results used as covariates. The significance threshold was evaluated with the formula *p* = 0.05/n (where n is the effective number of independent SNPs). The *p*‐value threshold for significance was set to ≈1.74 × 10^−8^. Results were summarized and visualized using the CMplot package (Version 4.5.1).^[^ ^55^ ^]^

### Membrane Permeability Assay

Whole plant material subjected to 300 mm NaCl treatment was placed in a 50‐mL centrifuge tube. After the addition of 15 mL of distilled water, the initial conductivity (E0) was measured with a conductivity meter (METTLER TOLEDO S700‐B). The centrifuge tube was shaken on a shaker at 200 rpm at room temperature for 30 min, and the conductivity (E1) was measured. The centrifuge tube was then incubated in boiling water at 100 °C for 20 min. After cooling, the final conductivity (E2) was measured. The relative conductivity was calculated using the formula: (E2‐E0)/(E1‐E0).

### Measurement of Anthocyanin Contents

The anthocyanin contents of different *S. bispinosa* accessions were measured as described previously.^[^ ^56^ ^]^ In brief, root samples harvested at the first compound leaf stage (≈15 days after germination) were weighed (FW, g) and ground in ethanol containing 1% HCl at a volume ratio of 1:1. After 2/3 volume of distilled water was added, the homogenate was transferred to an Eppendorf tube, mixed with 1 volume of chloroform, and centrifuged. The absorbance of the supernatant was measured at 535 nm (*A*_535_) using a spectrophotometer (TECAN‐Spark). The anthocyanin content was expressed as *A*_535_/FW. Each material was measured with three independent replicates.

### Functional Characterization of SbANS

To analyze the expression pattern of *SbANS*, root samples were harvested from plants at the first compound leaf stage (≈15 days after germination). Total RNA (≈2 µg) was extracted from the sample and reverse transcribed in a 20‐µL reaction mixture containing EasyScript cDNA Synthesis SuperMix (TRANSGEN Biotech). qRT‐PCR was performed using 1‐µL samples as templates to analyze *SbANS* transcript levels with the primers (F: 5ʹ‐CCCGACGCTATTCTTATGC‐3ʹ; R: 5ʹ‐CGCTGGCTCTGTTTCAGTT‐3ʹ). Three biological‐replicate samples were analyzed for each experiment. *Actin* (SbTA02G020930) was used as the internal control for qRT‐PCR with the primers (F: 5ʹ‐ACATTGTTCTTAGTGGTGGCT‐3ʹ; R: 5ʹ‐ATATTCACCCTTAGATATCCACAT‐3ʹ). To validate the function of *SbANS*, we constructed an expression vector (WMV084 backbone) containing the *SbANS* genomic DNA from *S. bispinosa* accession SbTA02 driven by the *35S* promoter and fused to the GUS reporter gene (35S::*SbANS*‐gDNA‐GUS). Transgenic soybean (*Glycine max* accession DN50) lines were generated by Wimi Biotechnology Company. Genotype screening was performed using 269‐bp vector‐specific primers targeting the *Bar* resistance gene (F: 5ʹ‐CCATCGTCAACCACTACATCGAGACA‐3ʹ; R: 5ʹ‐ CTTCAGCAGGTGGGTGTAGAGCGT‐3ʹ). Additionally, histochemical GUS staining with a GUS staining kit (SL7160, Coolaber) further verified successful *SbANS* expression in positive transgenic lines.

### RNA‐Seq

Total RNA samples were extracted from the root tissues of SbTA02 and SbSA44 plants at different time points under 300 mm NaCl treatment (0, 5, 10, 30, 60 min, and 24 h). Libraries were constructed for next‐generation sequencing using the MGISEQ sequencing platform. The RNA‐seq data were analyzed using Hisat2^[^ ^57^ ^]^ and Cufflinks. All acquired transcriptome data were assessed using FPKM values. Differential expression analysis was performed using the DESeq2 package.^[^ ^58^ ^]^

### Statistical Analysis

All measurements were expressed as the mean ± s.d. Statistical differences between two groups were evaluated using two‐tailed Student's *t*‐test with Excel software. The level of statistical significance of differences in mean was determined by *P*‐values, denoted here as follows: ^*^ *p* < 0.05, ^**^ *p* < 0.01, and ^***^ *p* < 0.001. Statistical differences among multiple comparisons are performed with a one‐way ANOVA using Tukey's multiple comparisons test at a 99.9% confidence interval. The sample size for each experiment and the corresponding *p*‐values were clearly indicated in the figure legends.

## Conflict of Interest

The authors declare no conflict of interest.

## Author Contributions

G.H., X.W., C.L., and K.H. contributed equally to this work. G.H. and X.C. conceived and designed the project. G.H., X.W., and H.L. conducted the genome assembly and performed bioinformatic analysis. C.L., K.H., X.H., C.Y., F.W., and S.Z. performed experiments. G.L. and Y.J. collected the germplasm. G.H. wrote the manuscript with assistance from X.C., X.D., and X.S.

## Supporting information



Supporting Information

Supplemental Tables

## Data Availability

The PacBio HiFi reads, Hi‐C data, next‐generation sequencing reads at the whole‐genome level, and RNA‐seq datasets generated in this study have been deposited in NCBI BioProject database under BioProject PRJNA1178852 and CNCB under BioProject PRJCA039464 (accession nos. CRR1787906 to CRR1787956). The resequencing reads from the S. *bispinosa* accessions generated in this study are available at NCBI under BioProject PRJNA1178855 and CNCB under BioProject PRJCA039494 (accession nos. CRR1787991 to CRR1788041). The CENH3 ChIP reads for SbTA02 and SbSA44 were deposited in NCBI under BioProject PRJNA1178880 (accession nos. SRR33324347 to SRR33324350) and CNCB under BioProject PRJCA039494 (accession nos. CRR1788416 to CRR1788419). The genome sequences and annotation files are available at the Genome Warehouse in CNCB under PRJCA039464 (accession nos. GWHGDGG00000000.1 for SbTA02 and GWHGDGH00000000.1 for SbSA44) and GitHub (https://github.com/huanggai/Sesbania‐bispinosa‐genome.git).
