# Supplementary material for: Genomic Variation Underpins Genetic Divergence and Differing Salt Resilience in Sesbania bispinosa
Source: Adv Sci (Weinh). 2025 May 29;12(32):e02600. doi: 10.1002/advs.202502600 (PMC12407362; doi:10.1002/advs.202502600)
Supplement: Supplementary file 1 — Supporting Information [file ADVS-12-e02600-s001.docx]

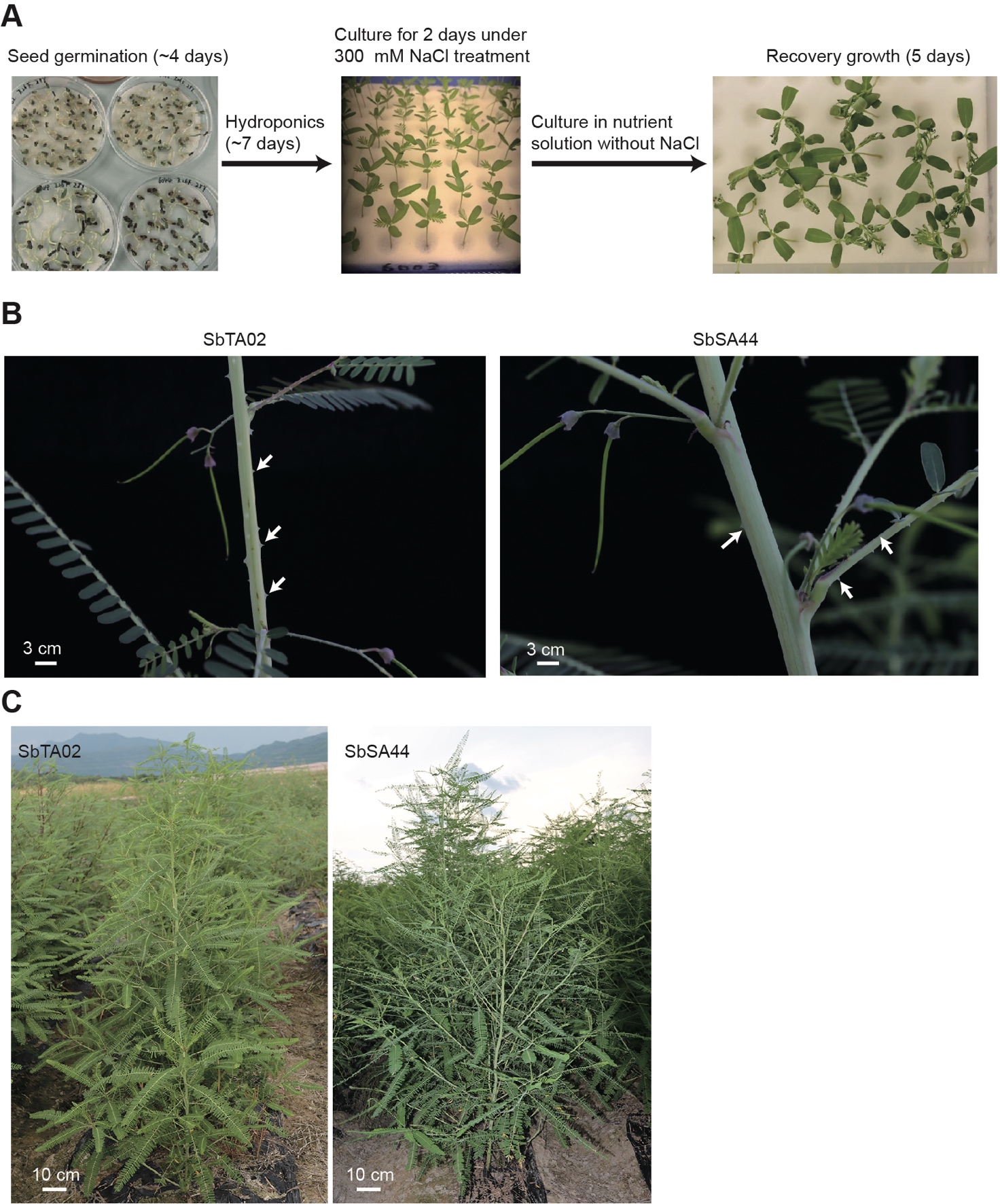


**Figure S1. The salt-tolerant evaluation system and morphological variations for *S. bispinosa*. A)** The salt-tolerant evaluation system for *S. bispinosa*. **B-C)** Plant morphology for salt-tolerant accession SbTA02 and salt-sensitive accession SbSA44. The spine grown on the stem and petioles was drawn with white arrows.


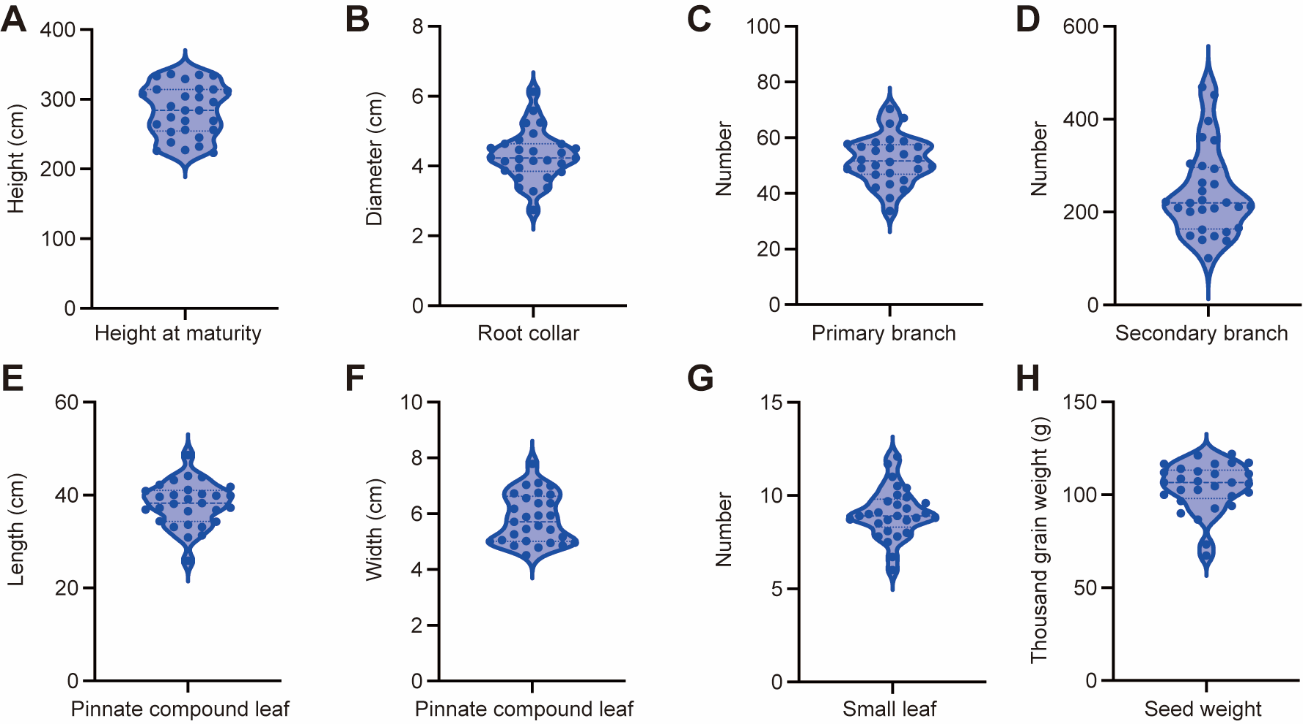


**Figure S2**. **Morphological variation among *S. bispinosa* accessions.** Violin plots showing the distribution of eight morphological traits across accessions: **A)** plant height at maturity, **B)** root collar diameter, **C)** number of primary branches, **D)** number of secondary branches, **E)** pinnate compound leaf length, **F)** pinnate compound leaf width, **G)** number of small leaves, and **H)** thousand-grain weight. The width of each violin indicates the density of data points at different values.


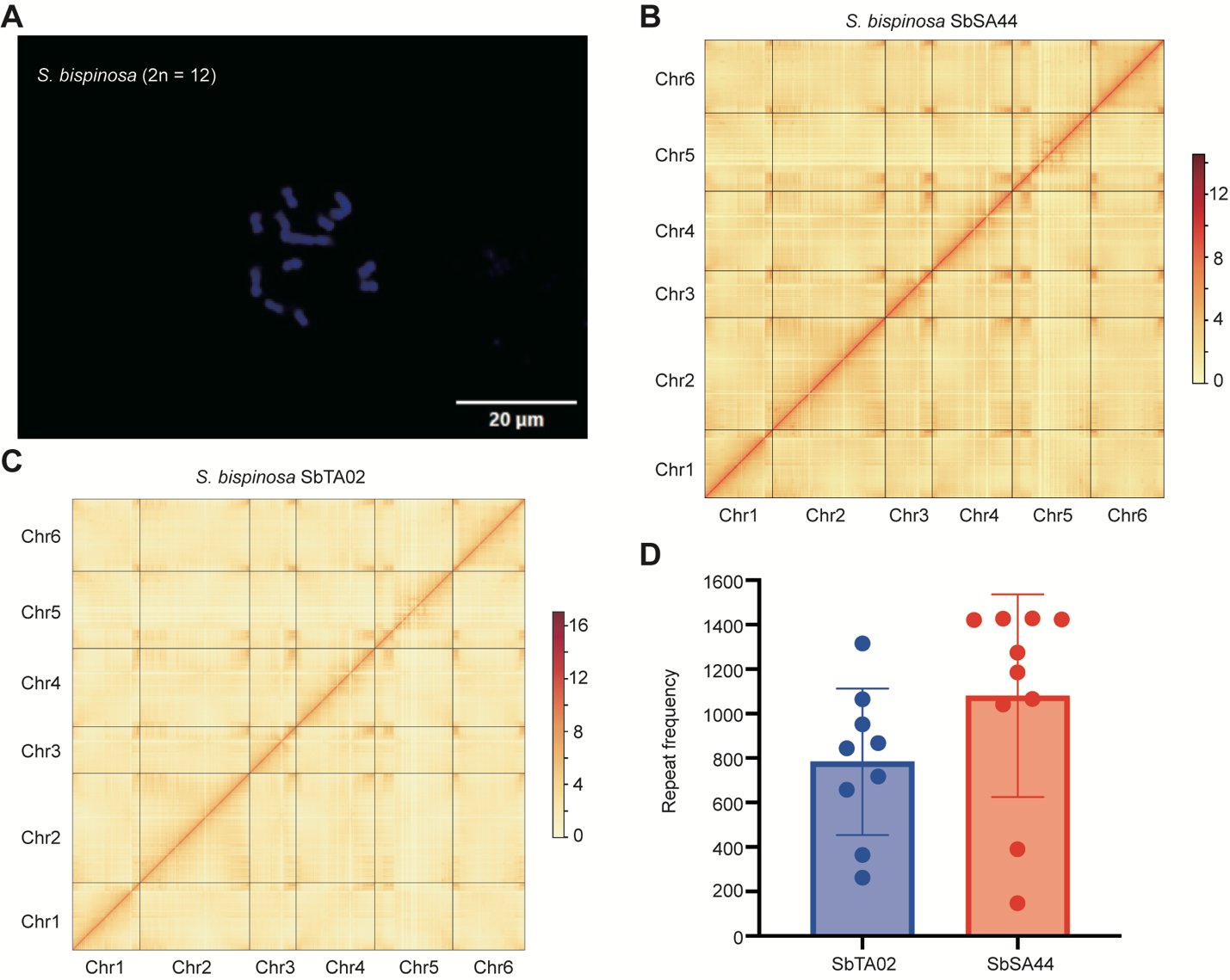


**Figure S3. Karyotyping and genomic analysis for two *S. bispinosa* genomes. A)** Karyotyping analysis revealed 6 pairs of chromosomes in diploid *S. bispinosa*. **B-C)** The Hi-C contact maps for the SbSA44 (**B**) and SbTA02 (**C**) genome. The strongest interaction signals (dark red color) clustered at the diagonal and end of each chromosome. **D)** Repeat frequency for telomeric repeats. The 10-kb chromosomal end sequences were used for telomeric repeat analysis.


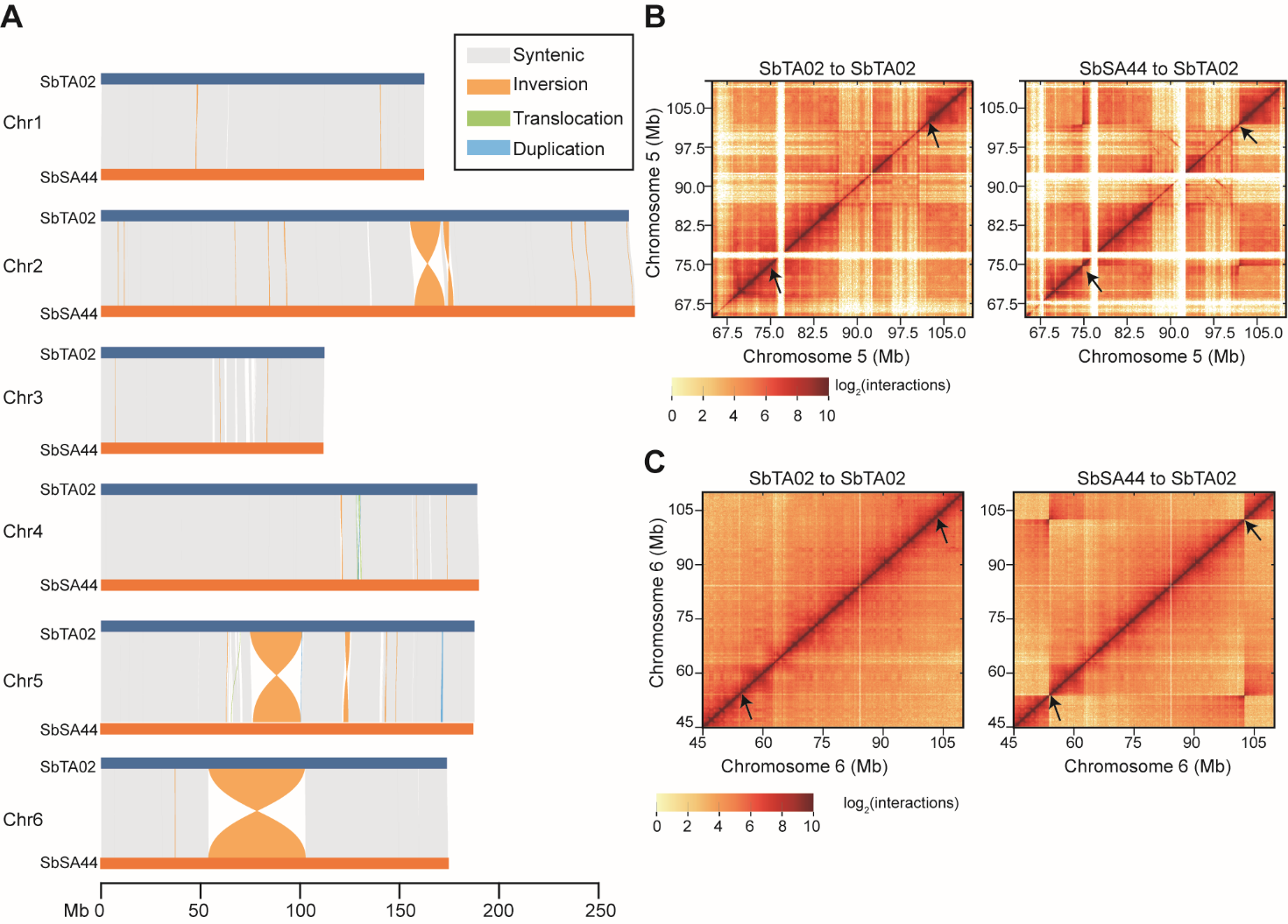


**Figure S4. Genome variation for two *S. bispinosa* genomes. A)** Genome variation between SbSA44 and SbTA02 genomes. **B-C)** Hi-C data validate the border of two large inversions in chromosomes 5 (**B**) and 6 (**C**) between SbSA44 and SbTA02 genomes. The upper heatmap shows a chromatin interaction matrix that maps Hi-C data from SbTA02 against the SbTA02-genome (SbTA02 map to SbTA02), and maps Hi-C data from SbSA44 against the SbTA02-genome (SbSA44 map to SbTA02).

**
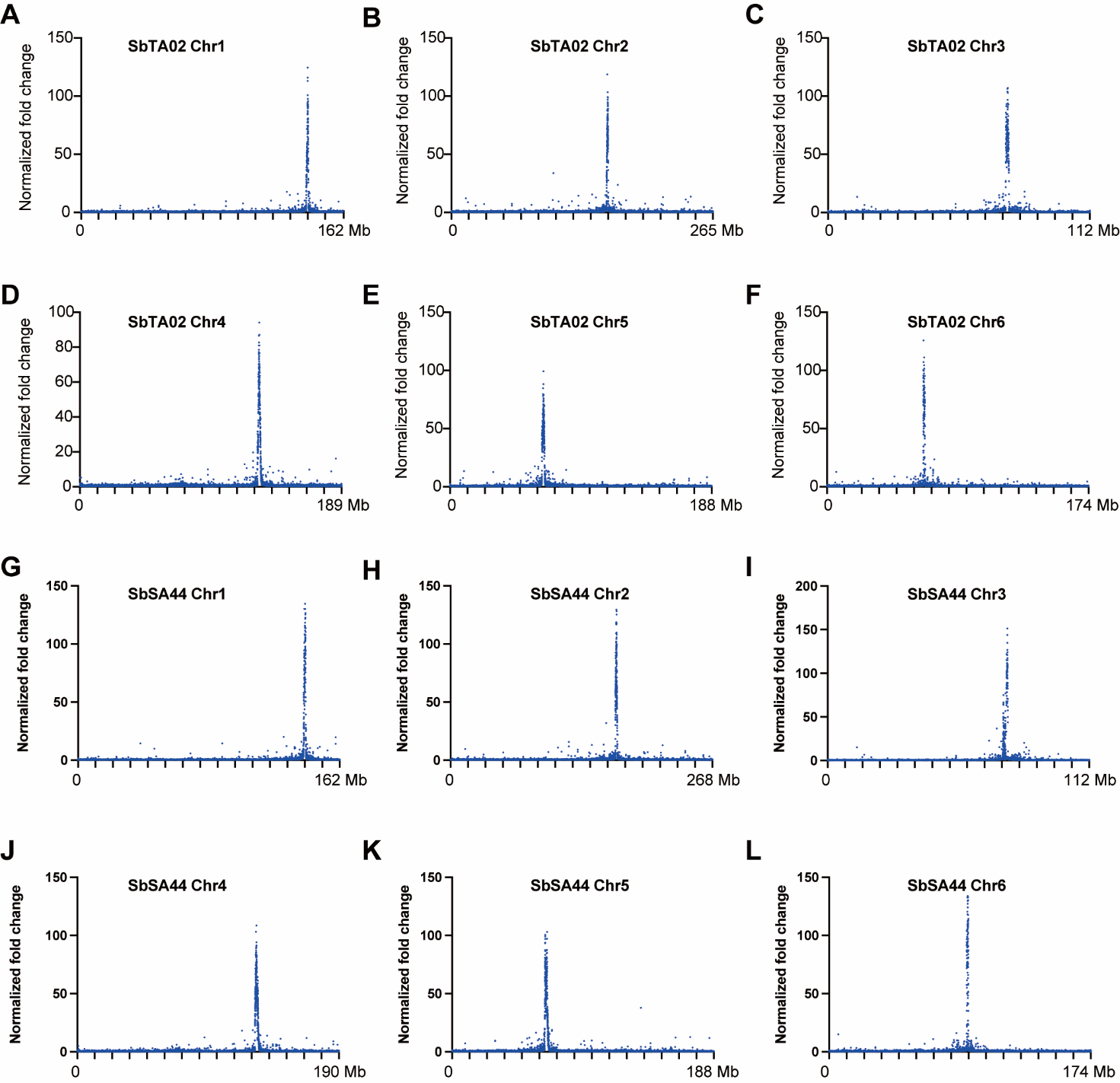
**

**Figure S5. CENH3 ChIP-seq analysis for two *S. bispinosa* genomes.** **A-F)** CENH3 ChIP-seq for SbTA02 across six chromosomes. **G-L)** CENH3 ChIP-seq for SbSA44 across six chromosomes. Normalized fold change refers to the ratio of normalized read counts between IP and input samples within 10-kb windows.


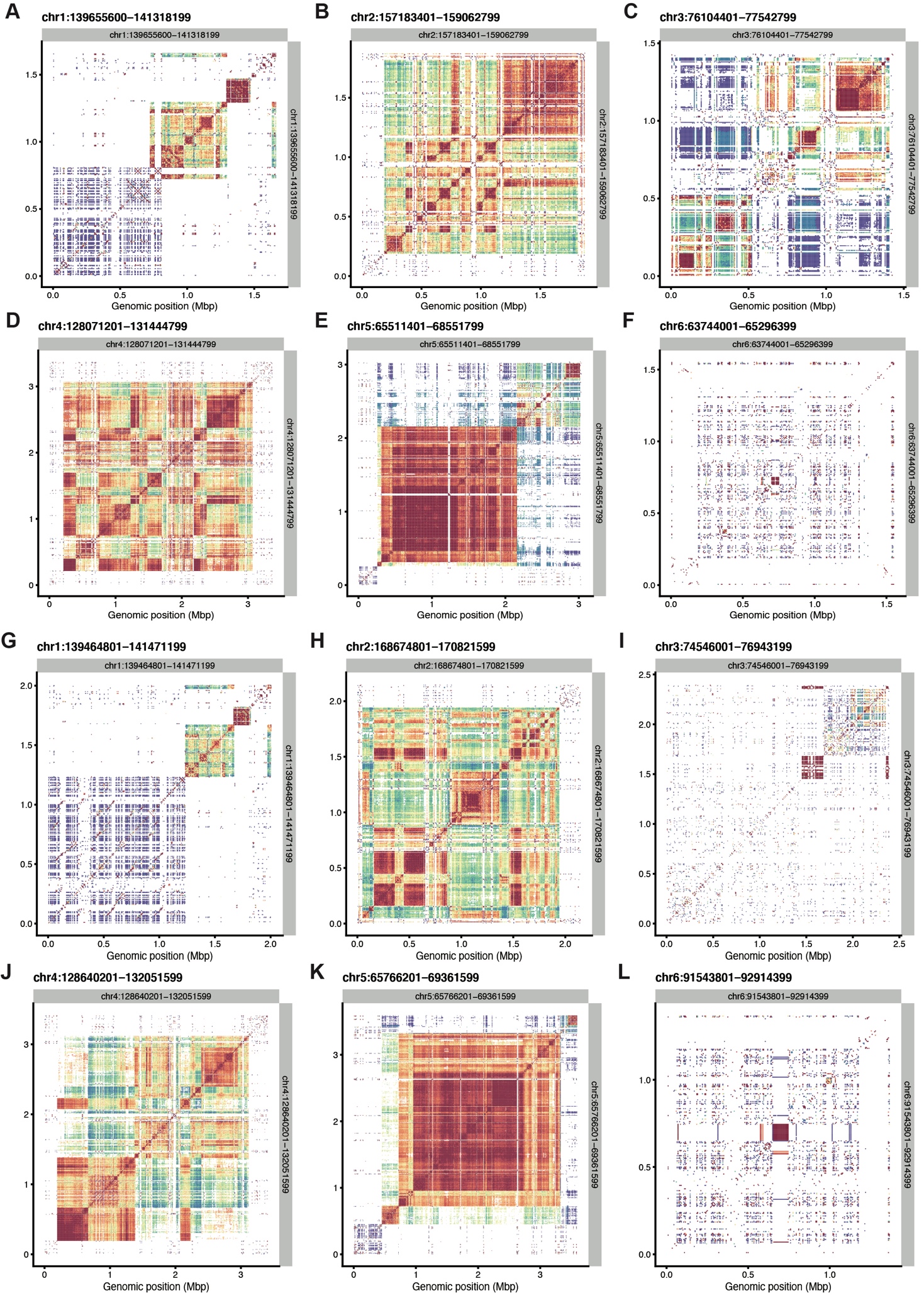


**Figure S6. Identity heatmap analysis of centromeric structures. A-F)** Identity heatmap analysis of Cen1 (**A**) to Cen6 (**F**) for *S. bispinosa* SbTA02. **G-L)** Identity heatmap analysis of Cen1 (**G**) to Cen6 (**L**) for *S. bispinosa* SbSA44.


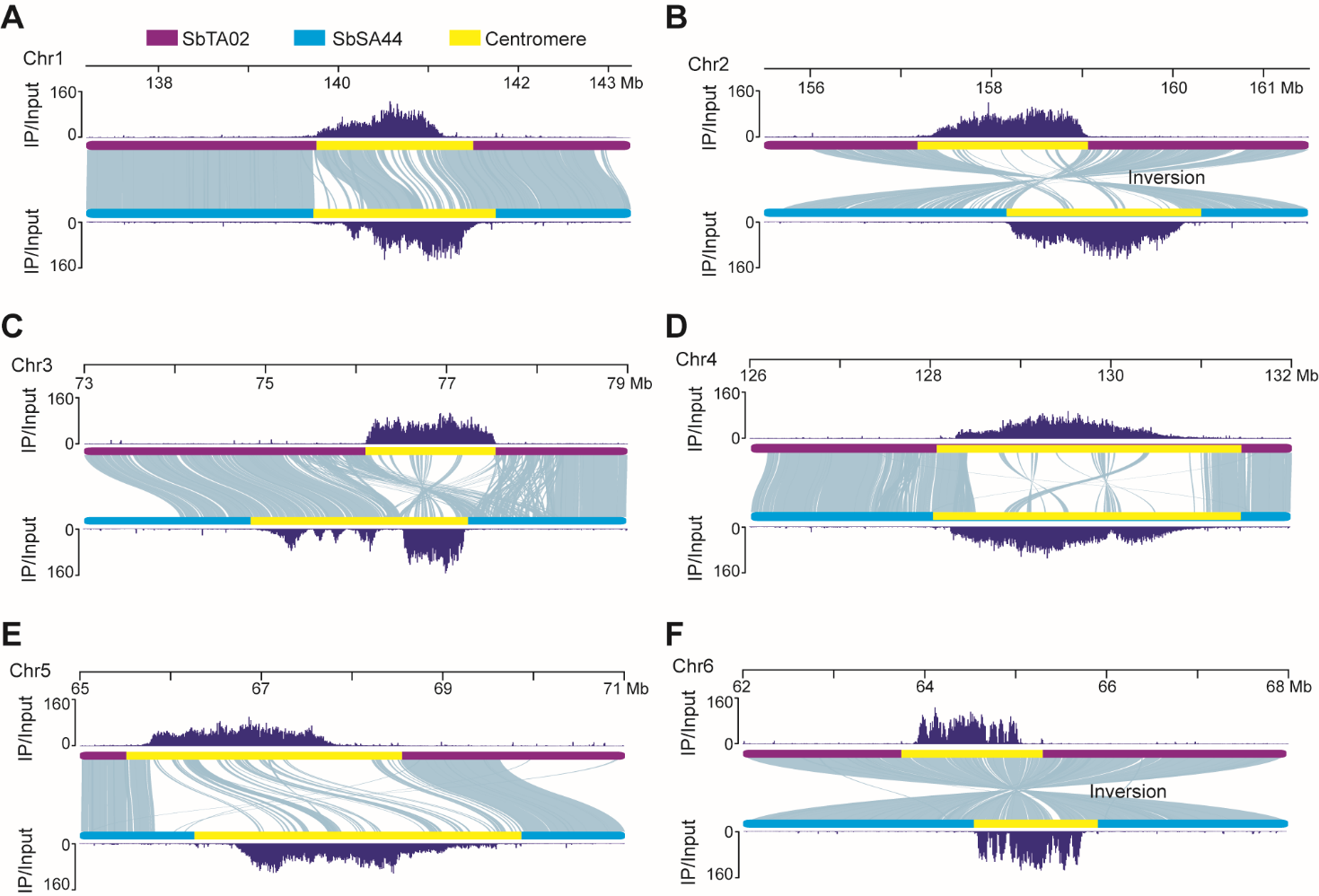


**Figure S7. Comparative analysis of centromeric sequences between the *S. bispinosa* genomes of SbTA02 and SbSA44. A-F)** Syntenic analysis of the centromeric and peri-centromeric regions of chromosomes 1 (**A**) to 6 (**F**).


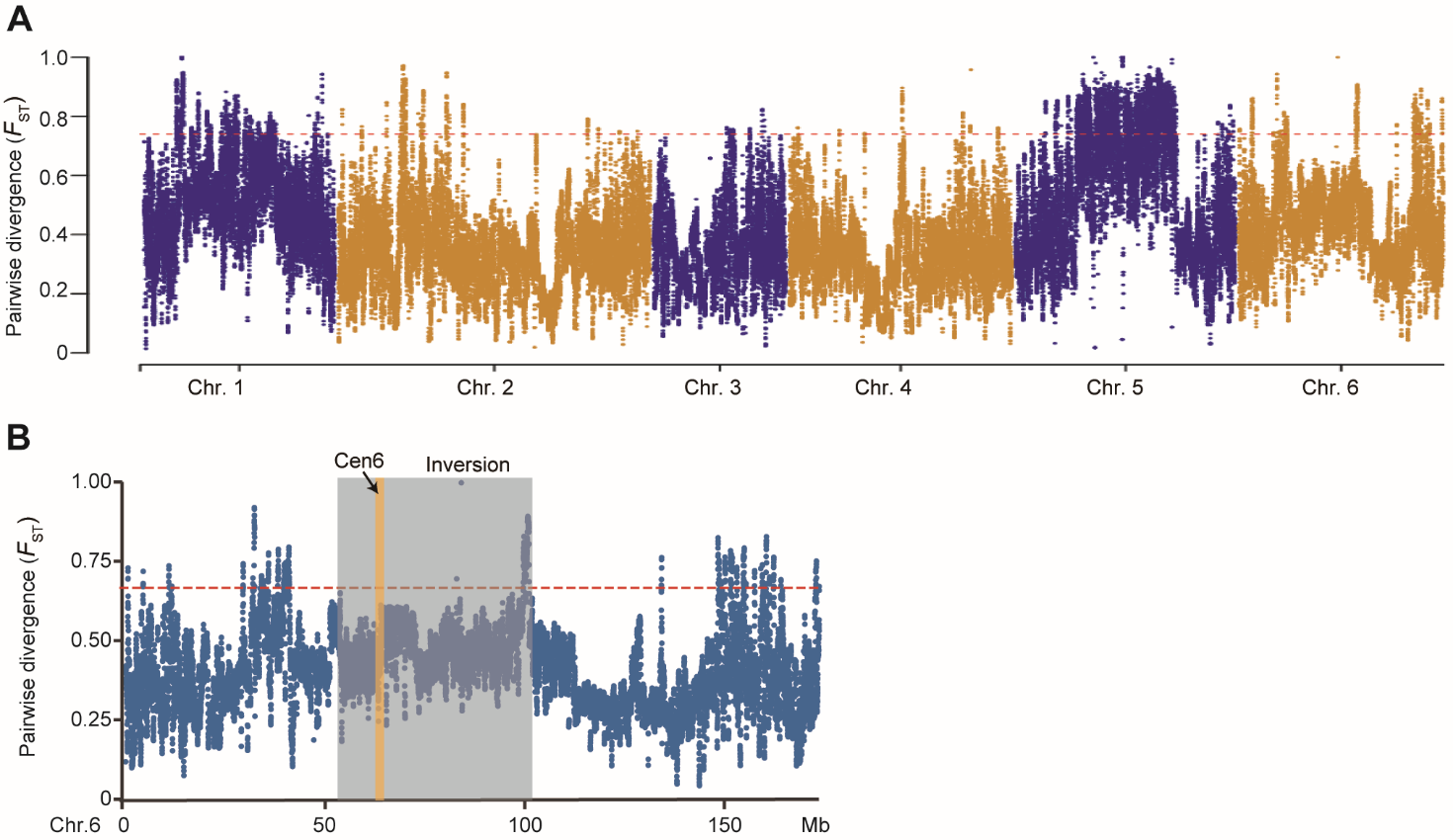


**Figure S8. Pairwise fixation statistic (F_ST_) for the AI and AC groups across six chromosomes (A) and on chromosome 6 (B).** Cen6, the centromere on chromosome 6.


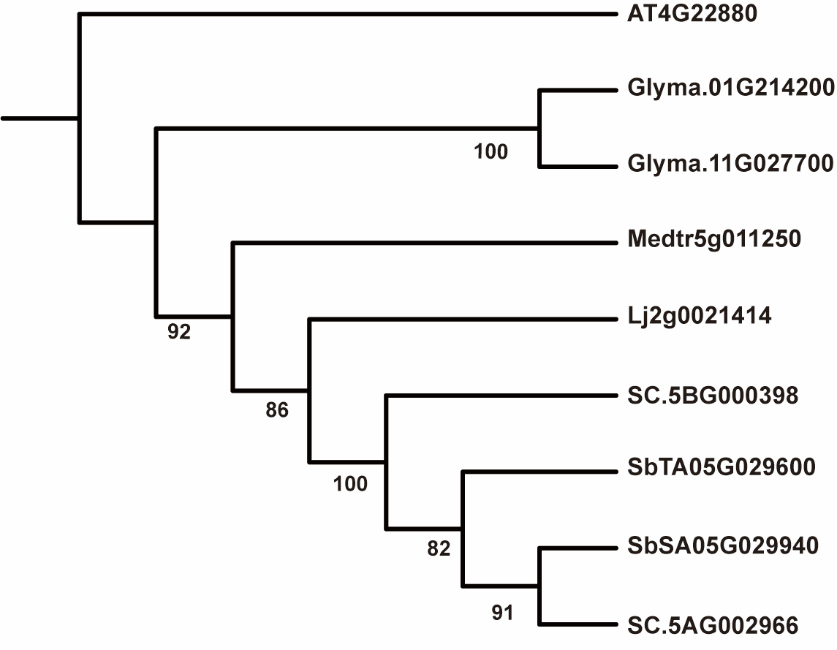


**Figure S9. Phylogenetic analysis of ANS/LDOX among representative plant species.** AT4G2280, Medtr5g011250, and Lj2g0021414 indicate the gene id from *Arabidopsis thaliana*, *Medicago truncatulat*, and *Lotus japonicus*, respectively. Glyma.01G214200 and Glyma.11G027700 represent gene id from *Glycine max*. SbTA05G029600, *S. bispinosa* SbTA02; SbSA05G029940, *S. bispinosa* SbSA44; SC.5AG002966 and SC.5BG000398 from tetraploid *S. cannabina*.

**
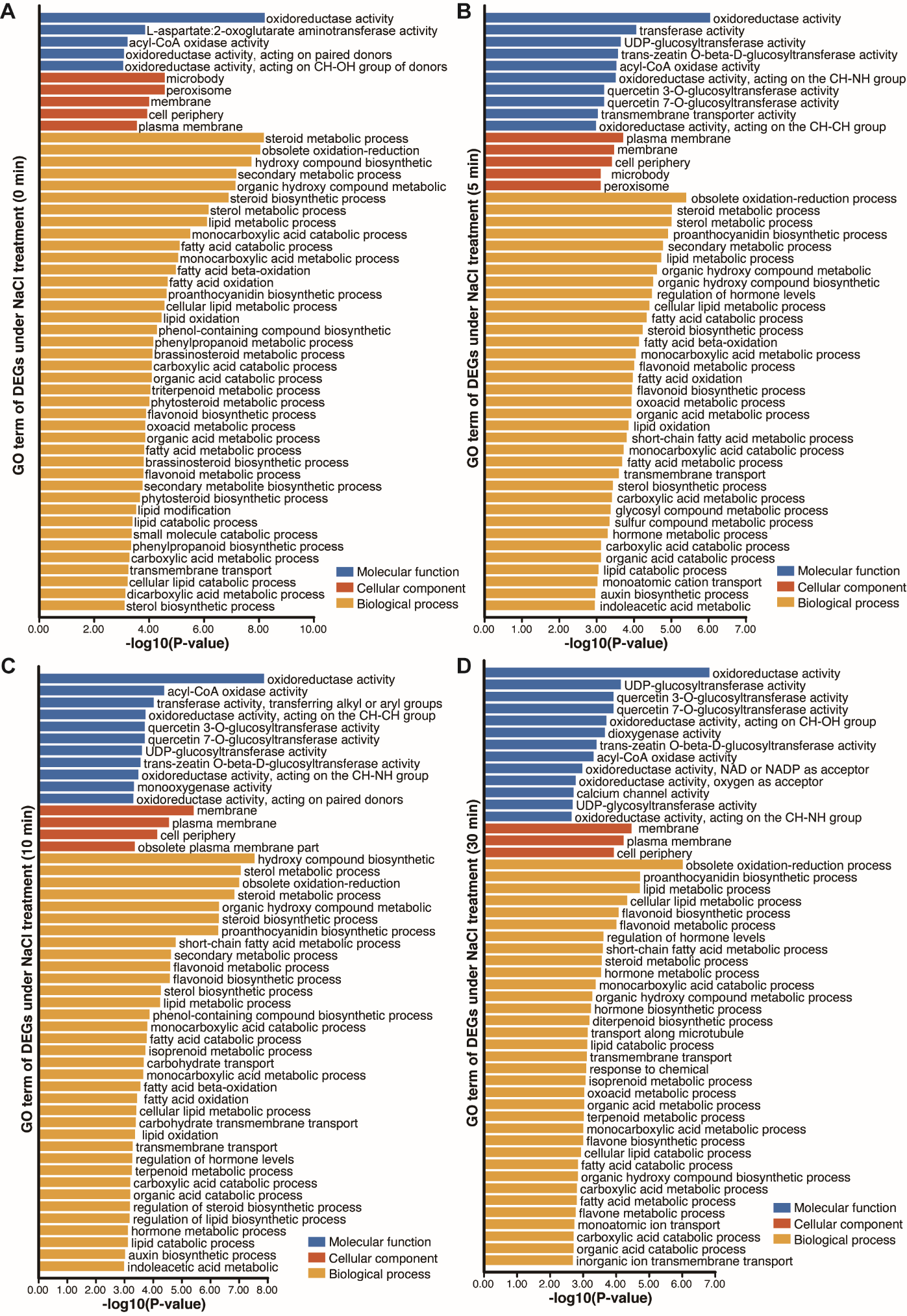
**

**Figure S10. Gene Ontology (GO) enrichment of differentially expressed genes (DEGs) between SbTA02 and SbSA44 under NaCl treatments at 0 min (A), 5 min (B), 10 min (C) and 30 min (D).**


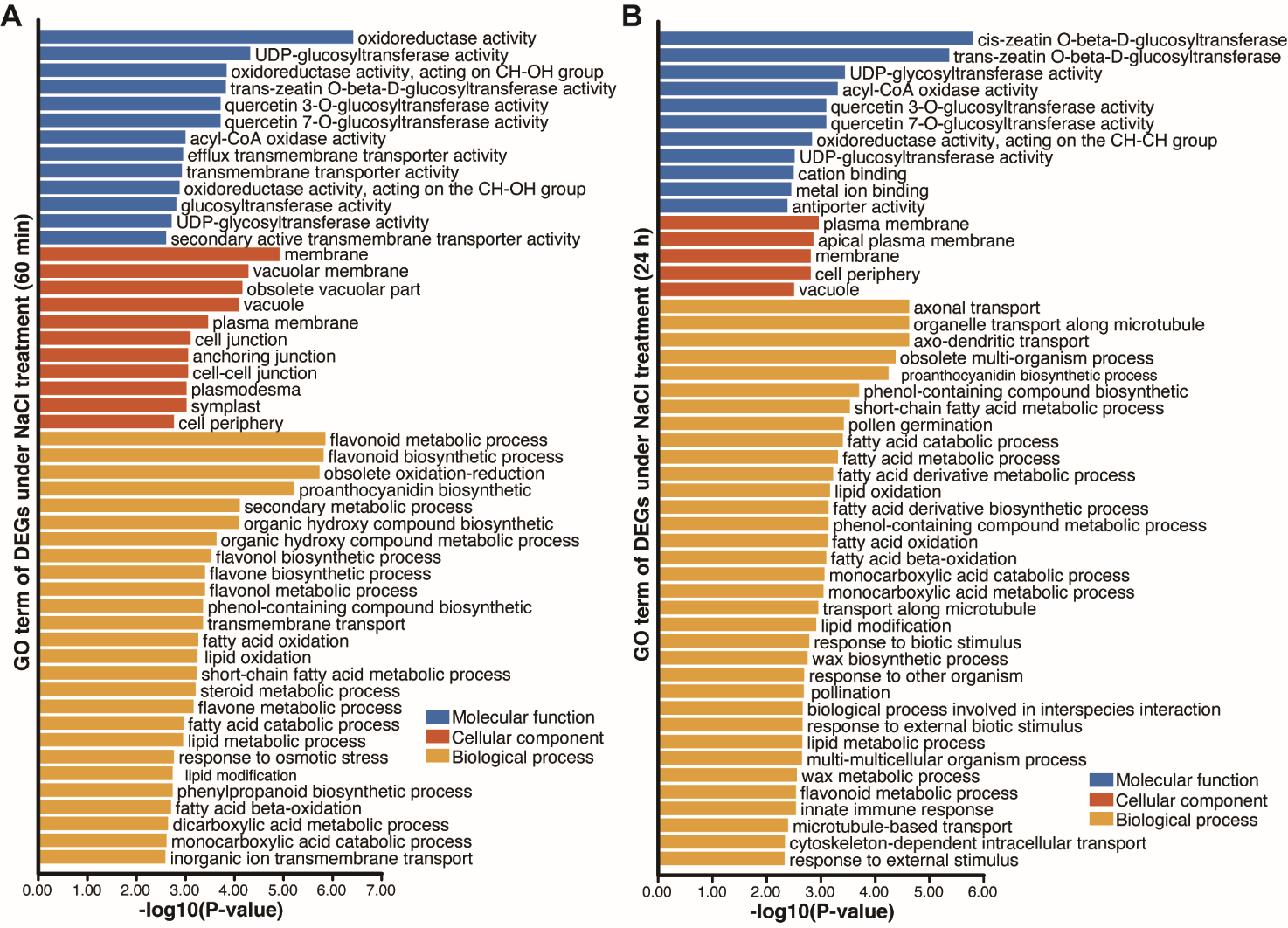


**Figure S11. Gene Ontology (GO) enrichment of differentially expressed genes (DEGs) between SbTA02 and SbSA44 under NaCl treatments at 60 min (A) and 24 h (B).**


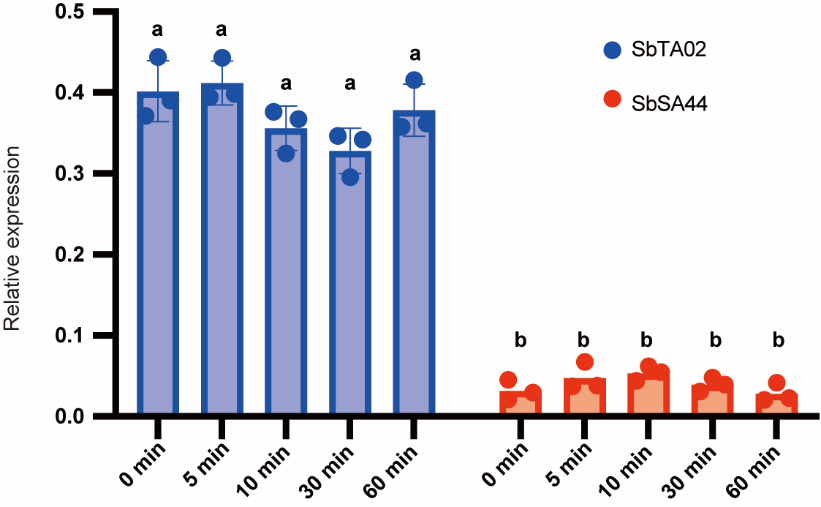


**Figure S12. Expression level of *SbANS* in root from salt-tolerant SbTA02 and salt-sensitive SbSA44 accessions.** Actin was used as the internal control for qRT–PCR data analysis. *n* = 3 independent experiments for each sample. A Two-Way ANOVA using multiple comparisons test at a 99.9% confidence interval was performed to identify significant differences between samples.


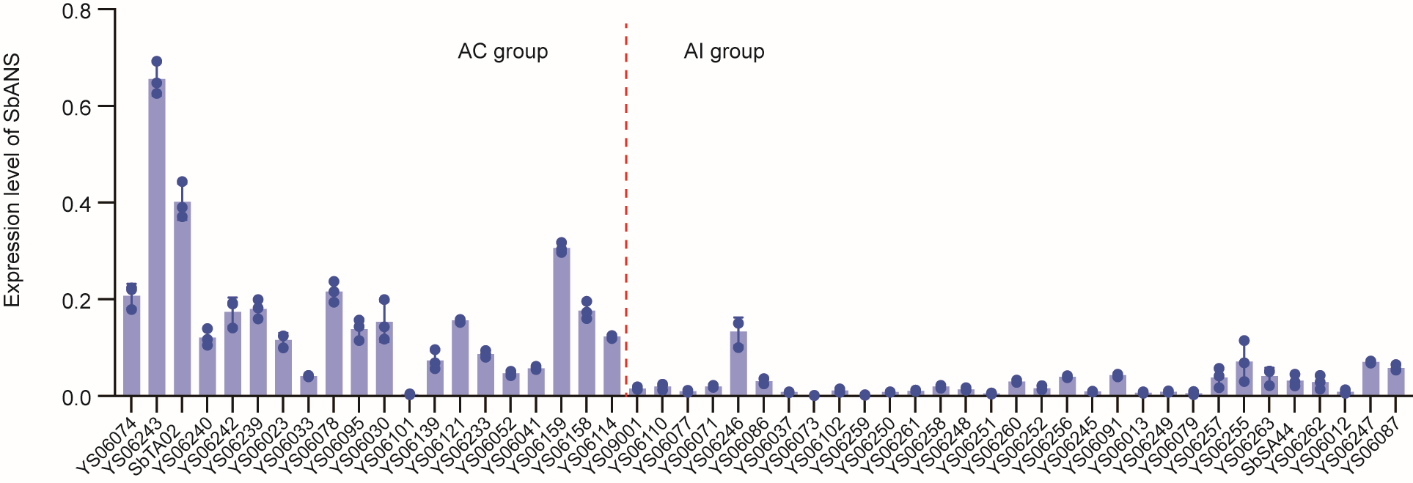


**Figure S13. Expression level of *SbANS* across *S. bispinosa* accessions.** Actin was used as the internal control for qRT–PCR data analysis. Data are presented as the mean ± s.d. *n* = 3 independent experiments for each sample.


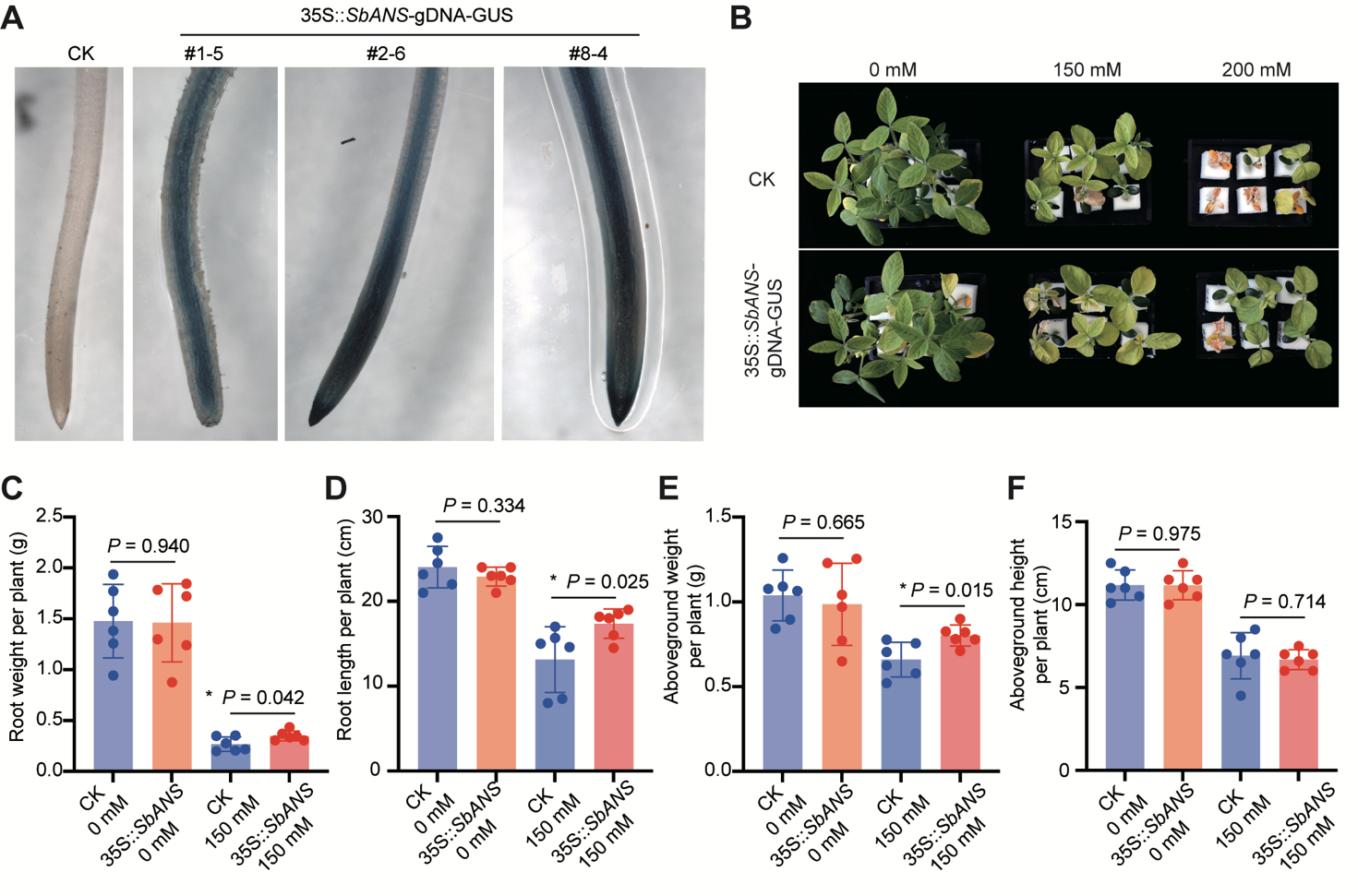


**Figure S14. Phenotypic analysis of transgenic soybean plants expressing *SbANS* from *S. bispinosa*. A)** Histochemical staining of control (CK) and transgenic (35S::*SbANS*-gDNA-GUS) plants using the GUS reporter gene. Blue color indicates GUS activity, confirming successful expression of the *SbANS* transgene in positive lines. **B)** Phenotypes of control (CK) and transgenic (35S::*SbANS*-gDNA-GUS) plants under NaCl treatment for 10 days. **C-F)** Quantitative measurements of growth parameters: (**C**) root weight, (**D**) root length, and (**E**) aboveground weight and (**F**) height. Data are presented as mean ± SE; asterisks indicate statistically significant differences (*P* < 0.05) (two-tailed Student’s *t*-test) between transgenic and control plants. *n* = 6 individual plants for each treatment.

**Supplementary tables**

**Table S1 Summary of sequencing data for *Sesbania bispinosa* genome.**

| Species | Platform | Total bases (bp) | Mean (bp) | Depth (×) |
| --- | --- | --- | --- | --- |
| *S. bispinosa* SbTA02 | PacBio HiFi reads | 62,920,785,361 | 15,043 | 57 |
|  | MGI reads for WGS | 79,320,061,500 | 150 | 72 |
|  | Hi-C library reads | 225,051,769,800 | 150 | 205 |
| *S. bispinosa* SbSA44 | PacBio HiFi reads | 58,630,947,683 | 17,132 | 53 |
|  | MGI reads for WGS | 83,559,890,400 | 150 | 76 |
|  | Hi-C library reads | 180,745,731,300 | 150 | 164 |

WGS, whole genome sequencing. MGI reads, reads generated on the DNBSEQ-T7 sequencing platform from MGI Technology.

**Table S2 Genome characteristics of two *Sesbania bispinosa* genomes.**

| Genomic feature | SbTA02 | SbSA44 |
| --- | --- | --- |
| Assembled genome size (Mb) | 1,090 | 1,094 |
| Number of contigs | 10 | 9 |
| Number of gaps | 4 | 3 |
| Number of >1 Mb centromeres | 6 | 6 |
| Number of telomeres | 9 | 10 |
| Annotated transposable elements (Mb) | 796 | 798 |
| Annotated transposable elements (%) | 73.0 | 72.9 |
| Total protein-coding genes | 48,835 | 48,575 |
| LAI (LTR assembly index) score | 17.3 | 16.7 |
| Complete BUSCOs (%) | 99.2 | 99.2 |
| Consensus quality value (QV) | 55.2 | 51.8 |

**Table S3 Transposable elements of two *Sesbania bispinosa* genomes.**

| Class | Family | SbTA02 | | |  | SbSA44 | | |
| --- | --- | --- | --- | --- | --- | --- | --- | --- |
|  |  | Count | Size (bp) | % |  | Count | Size (bp) | % |
| LTR | *Copia* | 210,919 | 223,318,630 | 20.48 |  | 219,876 | 228,652,730 | 20.90 |
|  | *Gypsy* | 373,129 | 334,282,985 | 30.66 |  | 368,225 | 327,951,095 | 29.97 |
|  | unknown | 19,516 | 27,563,839 | 2.53 |  | 16,362 | 24,932,933 | 2.28 |
| TIR | CACTA | 59,363 | 24,280,750 | 2.23 |  | 64,459 | 26,979,999 | 2.47 |
|  | Mutator | 100,197 | 39,998,818 | 3.67 |  | 109,544 | 46,736,161 | 4.27 |
|  | PIF Harbinger | 20,789 | 5,248,375 | 0.48 |  | 20,038 | 5,198,936 | 0.48 |
|  | Tc1 Mariner | 17,604 | 4,907,409 | 0.45 |  | 18,052 | 5,053,635 | 0.46 |
|  | hAT | 78,201 | 25,043,981 | 2.30 |  | 78,109 | 25,897,920 | 2.37 |
| Non-LTR | LINE element | 2,020 | 941,981 | 0.09 |  | 2,183 | 1,249,269 | 0.11 |
| Non-TIR | Helitron | 206,881 | 70,122,927 | 6.43 |  | 208,868 | 73,938,309 | 6.76 |
| Others | - | 143,193 | 40,426,272 | 3.71 |  | 106,294 | 31,370,016 | 2.87 |
| **Total** | **-** | **1,231,812** | **796,135,967** | **73.03** |  | **1,212,090** | **797,997,850** | **72.94** |

**Table S4 Location of centromeric regions from CENH3 ChIP-seq data for two *Sesbania bispinosa* genomes.**

| Name | SbTA02 | SbSA44 |
| --- | --- | --- |
| Cen1 | chr1:139655600-141320818 | chr1:139464801-141471199 |
| Cen2 | chr2:157183401-159062799 | chr2:168674801-170821599 |
| Cen3 | chr3:76104401-77542799 | chr3:74546001-76943199 |
| Cen4 | chr4:128071201-131444799 | chr4:128640201-132051599 |
| Cen5 | chr5:65511401-68568606 | chr5:65766201-69429470 |
| Cen6 | chr6:63744001-65296399 | chr6:91543801-92914399 |

**Table S5 Statistics of genome-sequencing data for *S. bispinosa* accessions.**

| **Accession name** | **No. of clean reads** | **Length (bp)** | **Total bases (bp)** | **Depth** | **No. of mapped reads** | **Mapping rate** |
| --- | --- | --- | --- | --- | --- | --- |
| YS06044/SbSA44 | 557,065,936 | 150 | 83,559,890,400 | 76.64 | 522,714,970 | 93.83% |
| YS06002/SbTA02 | 528,800,410 | 150 | 79,320,061,500 | 72.76 | 522,171,092 | 98.75% |
| YS06159 | 417,873,598 | 150 | 62,681,039,700 | 57.49 | 383,701,738 | 91.82% |
| YS06037 | 267,744,000 | 150 | 40,161,600,000 | 36.84 | 261,685,012 | 97.74% |
| YS06012 | 223,006,274 | 150 | 33,450,941,100 | 30.68 | 217,573,138 | 97.56% |
| YS06258 | 218,567,178 | 150 | 32,785,076,700 | 30.07 | 206,682,474 | 94.56% |
| YS06246 | 215,537,504 | 150 | 32,330,625,600 | 29.66 | 200,793,516 | 93.16% |
| YS06249 | 213,440,572 | 150 | 32,016,085,800 | 29.37 | 198,691,600 | 93.09% |
| YS06023 | 208,210,030 | 150 | 31,231,504,500 | 28.65 | 191,604,628 | 92.02% |
| YS06030 | 207,841,132 | 150 | 31,176,169,800 | 28.6 | 204,323,554 | 98.31% |
| YS06073 | 203,386,758 | 150 | 30,508,013,700 | 27.98 | 182,743,876 | 89.85% |
| YS06041 | 198,051,058 | 150 | 29,707,658,700 | 27.25 | 194,654,180 | 98.28% |
| YS06071 | 188,732,480 | 150 | 28,309,872,000 | 25.97 | 166,102,168 | 88.01% |
| YS06251 | 167,094,846 | 150 | 25,064,226,900 | 22.99 | 159,530,290 | 95.47% |
| YS06245 | 160,177,534 | 150 | 24,026,630,100 | 22.04 | 151,709,996 | 94.71% |
| YS06013 | 149,007,850 | 150 | 22,351,177,500 | 20.5 | 145,685,240 | 97.77% |
| YS06052 | 144,972,388 | 150 | 21,745,858,200 | 19.95 | 137,637,454 | 94.94% |
| YS09001 | 144,303,408 | 150 | 21,645,511,200 | 19.85 | 137,762,246 | 95.47% |
| YS06261 | 143,270,842 | 150 | 21,490,626,300 | 19.71 | 138,329,452 | 96.55% |
| YS06102 | 141,356,014 | 150 | 21,203,402,100 | 19.45 | 135,308,018 | 95.72% |
| YS06101 | 135,257,322 | 150 | 20,288,598,300 | 18.61 | 129,645,384 | 95.85% |
| YS06079 | 132,784,310 | 150 | 19,917,646,500 | 18.27 | 126,319,704 | 95.13% |
| YS06087 | 129,796,454 | 150 | 19,469,468,100 | 17.86 | 123,205,354 | 94.92% |
| YS06256 | 129,514,928 | 150 | 19,427,239,200 | 17.82 | 123,041,556 | 95.00% |
| YS06262 | 113,608,094 | 150 | 17,041,214,100 | 15.63 | 108,009,872 | 95.07% |
| YS06114 | 111,444,418 | 150 | 16,716,662,700 | 15.33 | 107,045,086 | 96.05% |
| YS06247 | 109,163,620 | 150 | 16,374,543,000 | 15.02 | 104,300,262 | 95.54% |
| YS06260 | 108,445,766 | 150 | 16,266,864,900 | 14.92 | 103,758,962 | 95.68% |
| YS06110 | 99,457,834 | 150 | 14,918,675,100 | 13.68 | 95,731,348 | 96.25% |
| YS06240 | 98,570,600 | 150 | 14,785,590,000 | 13.56 | 95,000,872 | 96.38% |
| YS06121 | 97,818,832 | 150 | 14,672,824,800 | 13.46 | 93,550,114 | 95.64% |
| YS06242 | 97,245,590 | 150 | 14,586,838,500 | 13.38 | 92,738,360 | 95.37% |
| YS06239 | 96,801,002 | 150 | 14,520,150,300 | 13.32 | 90,140,228 | 93.12% |
| YS06086 | 96,064,002 | 150 | 14,409,600,300 | 13.22 | 86,645,072 | 90.20% |
| YS06257 | 95,500,522 | 150 | 14,325,078,300 | 13.14 | 89,033,638 | 93.23% |
| YS06263 | 95,482,116 | 150 | 14,322,317,400 | 13.14 | 91,035,180 | 95.34% |
| YS06233 | 91,460,236 | 150 | 13,719,035,400 | 12.58 | 83,718,462 | 91.54% |
| YS06077 | 91,180,644 | 150 | 13,677,096,600 | 12.55 | 78,905,550 | 86.54% |
| YS06248 | 86,756,638 | 150 | 13,013,495,700 | 11.94 | 82,022,478 | 94.54% |
| YS06243 | 86,524,154 | 150 | 12,978,623,100 | 11.9 | 80,459,270 | 92.99% |
| YS06252 | 84,568,776 | 150 | 12,685,316,400 | 11.64 | 81,316,820 | 96.15% |
| YS06250 | 80,717,340 | 150 | 12,107,601,000 | 11.11 | 76,747,270 | 95.08% |
| YS06158 | 79,797,768 | 150 | 11,969,665,200 | 10.98 | 76,313,416 | 95.63% |
| YS06259 | 79,177,706 | 150 | 11,876,655,900 | 10.89 | 75,894,760 | 95.85% |
| YS06095 | 78,493,288 | 150 | 11,773,993,200 | 10.8 | 72,615,018 | 92.51% |
| YS06255 | 78,303,902 | 150 | 11,745,585,300 | 10.77 | 72,913,444 | 93.12% |
| YS06074 | 76,904,880 | 150 | 11,535,732,000 | 10.58 | 70,034,780 | 91.07% |
| YS06091 | 73,378,894 | 150 | 11,006,834,100 | 10.1 | 65,887,002 | 89.79% |
| YS06139 | 71,355,832 | 150 | 10,703,374,800 | 9.82 | 63,252,584 | 88.64% |
| YS06033 | 67,822,394 | 150 | 10,173,359,100 | 9.33 | 63,998,922 | 94.36% |
| YS06078 | 63,963,672 | 150 | 9,594,550,800 | 8.8 | 60,346,066 | 94.34% |
| **Average** | **-** | **-** | **-** | **20.6** | **-** | **94.28%** |
